# Modeling the human aging transcriptome across tissues, health status, and sex

**DOI:** 10.1111/acel.13280

**Published:** 2020-12-18

**Authors:** Maxim N. Shokhirev, Adiv A. Johnson

**Affiliations:** ^1^ Razavi Newman Integrative Genomics and Bioinformatics Core Salk Institute for Biological Studies La Jolla CA USA; ^2^ Adiv A. Johnson Tucson AZ USA

**Keywords:** age prediction, aging clock, machine learning, meta‐analysis, random forest, transcriptomics

## Abstract

Aging in humans is an incredibly complex biological process that leads to increased susceptibility to various diseases. Understanding which genes are associated with healthy aging can provide valuable insights into aging mechanisms and possible avenues for therapeutics to prolong healthy life. However, modeling this complex biological process requires an enormous collection of high‐quality data along with cutting‐edge computational methods. Here, we have compiled a large meta‐analysis of gene expression data from RNA‐Seq experiments available from the Sequence Read Archive. We began by reprocessing more than 6000 raw samples—including mapping, filtering, normalization, and batch correction—to generate 3060 high‐quality samples spanning a large age range and multiple different tissues. We then used standard differential expression analyses and machine learning approaches to model and predict aging across the dataset, achieving an *R* ^2^ value of 0.96 and a root‐mean‐square error of 3.22 years. These models allow us to explore aging across health status, sex, and tissue and provide novel insights into possible aging processes. We also explore how preprocessing parameters affect predictions and highlight the reproducibility limits of these machine learning models. Finally, we develop an online tool for predicting the ages of human transcriptomic samples given raw gene expression counts. Together, this study provides valuable resources and insights into the transcriptomics of human aging.

## Graphical abstract

In order to better understand aging, we comprehensively analyzed 3060 high‐quality human transcriptomic samples. These samples spanned diverse tissues as well as an age range of <1–107 years. Using machine learning, we demonstrate that genes which change their expression level over time are able to accurately predict age. We additionally show that health, sex, and tissue all impact which genes are utilized to predict age and explore the generalizability of transcriptomic aging clocks.

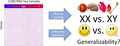

## 1 INTRODUCTION

In 2011, Bocklandt et al. demonstrated that sites of DNA methylation in saliva samples are able to predict human age (Bocklandt et al., 2011). They showed that a regression model could be trained to predict aging using just 88 loci that were selected based on correlation. Steve Horvath, who was a contributor to the study by Bocklandt et al. (2011), went on to design a more robust epigenetic age predictor in 2013 (Horvath, 2013). The predictive power of this revamped age estimator was quite high in various tissues, with prominent examples including whole blood (Pearson's correlation = 0.95), peripheral blood mononuclear cells (Pearson's correlation = 0.96), and occipital cortex (Pearson's correlation = 0.98). In other samples, such as adipose fat (Pearson's correlation = 0.65) or uterine endometrium (Pearson's correlation = 0.55), the predictive power was more moderate. The sites of methylated DNA used to estimate human age were referred to as an aging clock (Horvath, 2013). Earlier that same year, a separate epigenetic aging clock was developed by Hannum et al. This clock was specific to whole blood and was quite predictive (R = 0.96 for the primary cohort and R = 0.91 for the validation cohort; Hannum et al., 2013). Subsequent generations of epigenetic aging clocks—dubbed PhenoAge (Levine et al., 2018) and GrimAge (Lu et al., 2019)—were trained on longitudinal data and could therefore predict phenotypic age, time to morbidity, or onset of various diseases. These newer clocks (Levine et al., 2018; Lu et al., 2019) could predict the risk of disease, such as coronary heart disease and Alzheimer's disease, which were associated with significantly increased phenotypic age. This phenomenon, in the context of an epigenetic aging clock, is referred to as epigenetic age acceleration (Horvath & Raj, 2018).

Diverse data types besides methylated DNA can also be used to predict human age, such as RNA (Galkin et al., 2020). In the same previously mentioned study by Hannum et al. (2013), mRNA expression data from 488 individuals were used to create a whole blood transcriptomic aging clock with an *R* ^2^ value of 0.745. RNA in human peripheral blood was previously mined by Peters et al. to generate transcriptomic aging clocks in eight different cohorts. Depending on the cohort analyzed, the *R* ^2^ value ranged from 0.121 to 0.599. The number of samples used varied by cohort and varied from 513 to 2446 (Peters et al., 2015). By using an ensemble machine learning method to analyze transcriptomic datasets derived from dermal fibroblasts of 133 healthy human patients (age range of 1–94 years), Fleischer et al. uncovered a RNA signature that could predict human age with an increased accuracy (*R* ^2^ = 0.81; Fleischer et al., 2018). Repetitive elements from this same dataset were later used in conjunction with linear regression to create a more accurate clock with a *R* ^2^ of 0.93 (LaRocca et al., 2020). By analyzing 545 human skeletal muscle samples, a separate RNA clock was constructed using a deep feature selection model that achieved a Pearson correlation of 0.91 (Mamoshina et al., 2018). In a whole blood dataset involving 5221 adults, a microRNA clock was also built using elastic net regression that had a Pearson correlation of 0.65 in the replication set (Huan et al., 2018). Recently, we also demonstrated that protein abundance can be used to predict aging (Johnson et al., 2020). Thus, in addition to methylation and clinical data, aging clocks over varying accuracies can be constructed from transcriptomes, repeat elements, microRNAs, and protein abundance measures, revealing specific biological signatures across diverse data types. Various machine learning models can also be employed, including deep learning algorithms (Gialluisi et al., 2019; Zhavoronkov et al., 2019).

While biological age markers are highly desirable for their potential ability to quantify healthy vs. unhealthy aging, they can also teach us about what causes human aging. If a molecule can be used to predict a patient's age, it seems likely that such a molecule has an increased chance of being pertinent to the regulation of health span or life span. The same omics datasets used to generate aging clocks can also be thoroughly analyzed to learn more about molecular changes that occur with age. These molecular changes can themselves be investigated to unveil what functions and processes are the most profoundly altered between different age classes (Valdes et al., 2013). For example, the laboratory of Anne Brunet analyzed transcriptomes to discover that innate immune pathways become dysregulated with age in mice, African turquoise killifish, rats, and humans (Benayoun et al., 2019). Recent work spearheaded by Tony Wyss‐Coray demonstrated that deleterious lipid‐droplet‐accumulating microglia build up with age in mouse and human brains. Transcriptional profiling of these aberrant microglial cells revealed unique changes in various pathways, including innate inflammation (Marschallinger et al., 2020).

In the present study, we sought to determine whether or not an especially predictive transcriptomic aging clock could be generated. We additionally wanted to better understand the major changes that occur with human age on a RNA level. To do this, we employed machine learning, differential expression, and enrichment analyses in a large set of high‐quality, RNA‐Seq samples from diverse human tissues. In addition to creating an ultra‐predictive RNA aging clock, we additionally generate multiple tissue‐specific, sex‐specific, and health‐specific age predictors, explore the generalizability of aging clocks, and identify key genes and associated processes that change with age.

## 2 RESULTS

### 2.1 An integrated dataset of human transcriptomes

**FIGURE 1:**
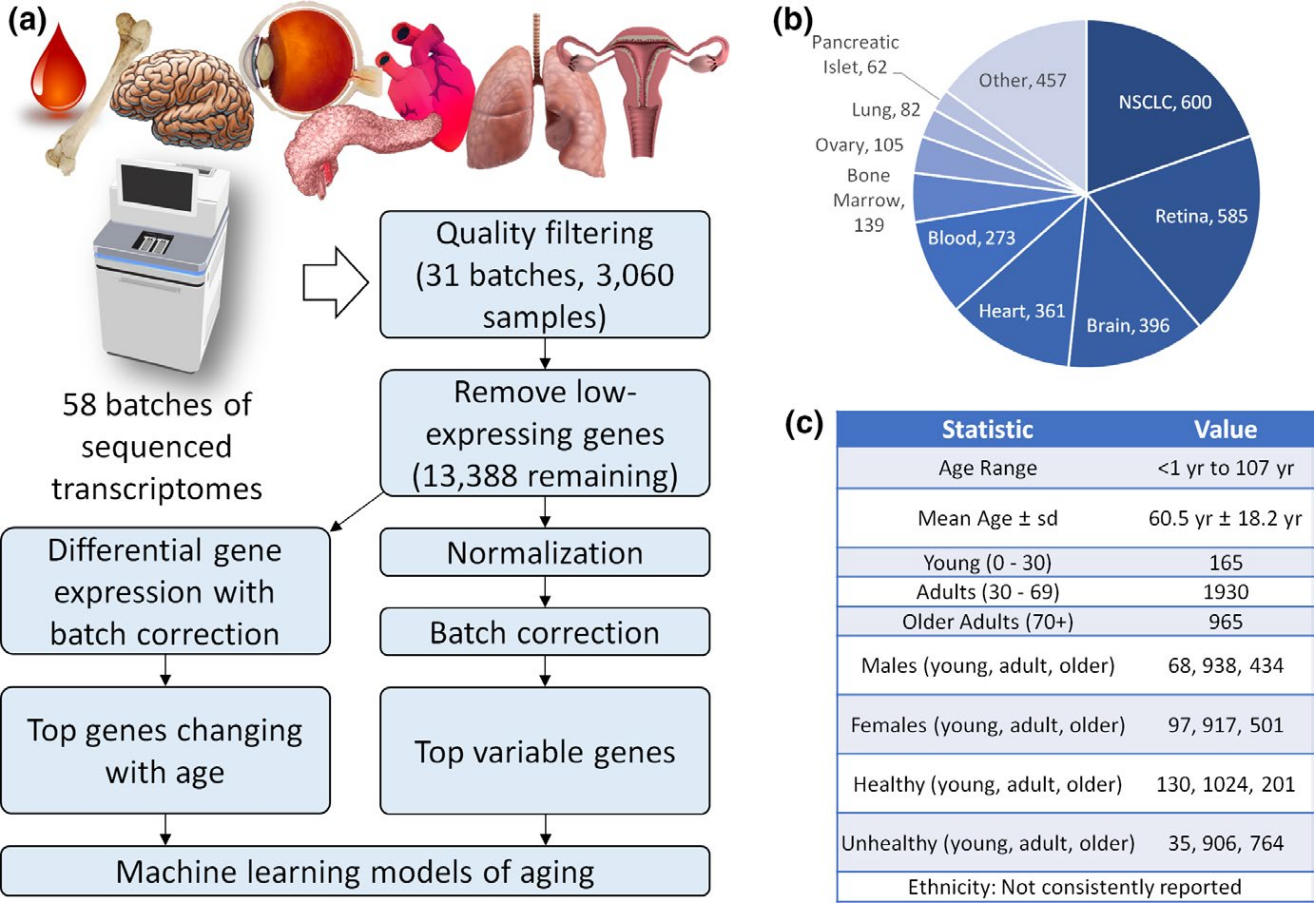
Building and integrating a computational model of the human aging transcriptome. (a) Analysis workflow demonstrating how 6471 raw RNA‐Seq samples were filtered into 3060 high‐quality samples. These high‐quality samples were then normalized, batch‐corrected, and utilized to identify genes that change with age and to develop predictive aging clocks. (b) Tissue distribution across samples. C) Summary of combined cohort statistics. NSCLC, non‐small‐cell lung carcinoma

In order to identify transcripts that are predictive of aging, we began by going through the literature and NCBI Sequence Read Archive (Leinonen et al., 2011) for publicly available RNA‐Seq datasets. Specifically, we collated human RNA‐Seq datasets that provide raw sequencing data and include sample information regarding health status and age. We then developed a bioinformatics pipeline for mapping, filtering, normalizing, and modeling the data in a systematic way (Figure 1a). Importantly, after mapping and counting raw reads across gene exons, we found a multi‐modal distribution of total counts among samples, which we used to remove low‐expressing samples (Figure S1a). In addition, since not all datasets have a clear aging signature in our models, batches that improve accuracy when left out of our machine learning steps were also removed (Figure S1b, Table S1). Finally, genes which had overall low average expression across the remaining samples were filtered (Figure S1c). Importantly, since batch effects produced the strongest signals among our combined datasets (Figure S2a–d), we included batch‐effect correction as an essential step to minimize technical noise (Figure S2e–h). This resulted in 3060 samples from 31 separate batches and more than 10 different tissues (Figure 1b). Our datasets show an age range of less than 1 year to 107 years and an average age of 60.5 ± 18.2 years (Figure 1c). We further divided the dataset into young <30 years, adult ≥30 but <70 years, and older adults ≥70 years as described below. Across these age groups, we observed a similar distribution of samples across sex and health status. Ethnicity was not considered as it was not reported in many studies (Figure 1c). Importantly, batches varied by health status, sex, age distribution, and sample count (Figure S3), highlighting the importance of an integrative aging model.

### 2.2 Human transcriptomes vary with age

**FIGURE 2:**
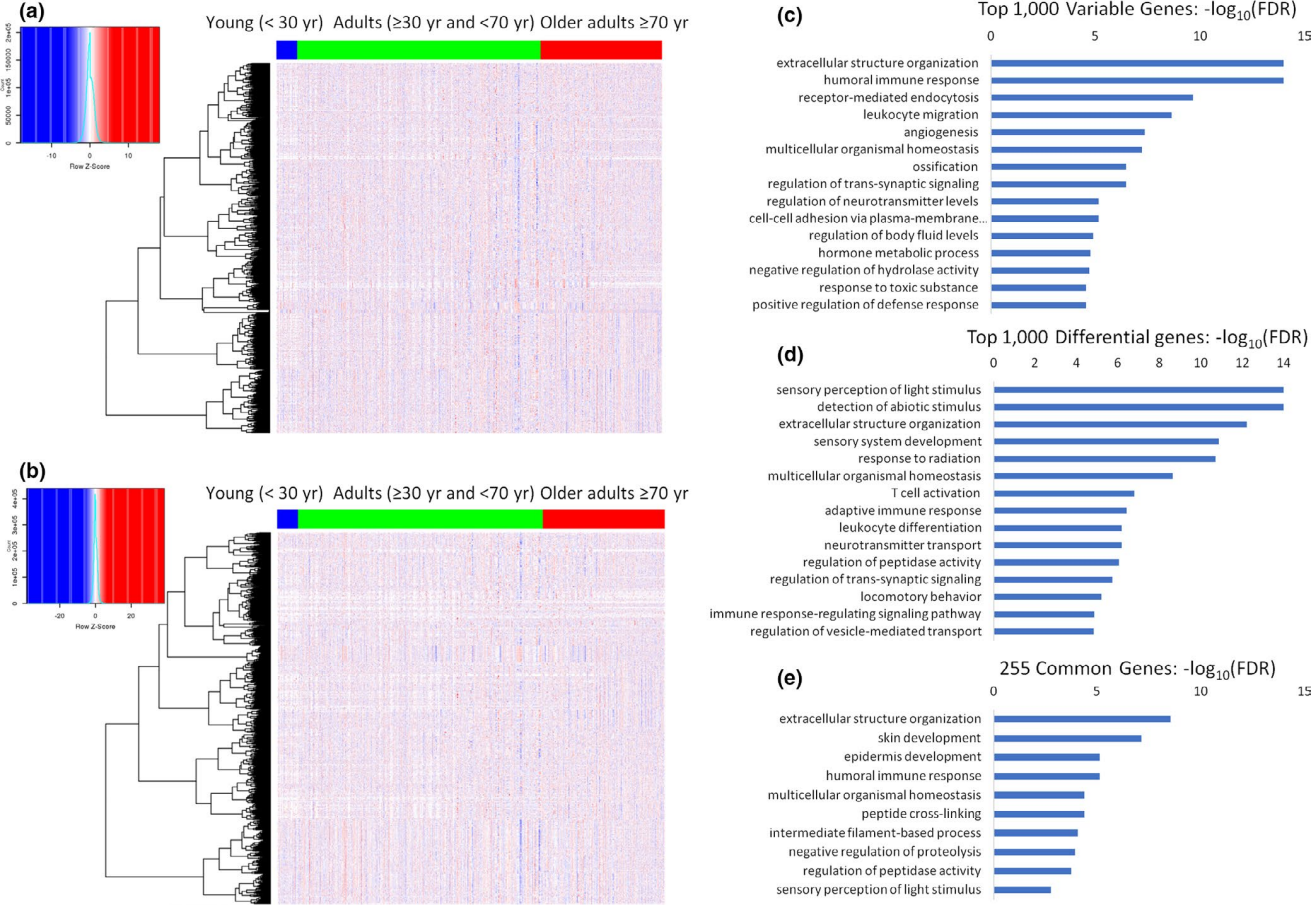
Genes change their expression with age. (a) Heatmap showing batch‐corrected normalized variable genes across ages. (b) Heatmap showing genes differentially changed between adults (30–69 years) and older adults (≥70 years). (c–e) Over‐representation analyses showing the top Gene Ontology terms identified as significant among the 1000 most variable genes (c), the 1000 most differential genes (d), and the 255 genes common to both sets (e)

After collecting and preprocessing the data, we identified the top 1000 variable genes across age. We then manually subdivided the samples into three age groups based on separation of the gene expression signatures in a clustered heatmap ordered by age (Figure 2a). While variable genes across age provide a general view into what biological changes are occurring, we also performed a differential expression analysis between the adult and older adult groups of samples, including the batch as a covariate in the modeling (Figure 2b). By performing a simple over‐representation analysis on the top 1000 variable genes, we identified many Gene Ontology (GO) biological processes that have significant overlap, including immune, signaling, homeostasis, and extracellular structure terms (Figure 2c). We also picked the top 1000 differentially expressed genes between these groups and performed a similar analysis to see which biological terms are over‐represented among them. While some terms showed a similar theme of the immune response/activation, homeostasis, and extracellular structure, there were additional neuronal/sensory terms which were not found to be significantly overlapped among the variable genes (Figure 2d). Of note, only 255 genes were shared between the top variable and top differential genes, which had significant overlap with extracellular structure organization, the humoral immune response, and homeostasis (Figure 2e). Complete lists of the top 1000 variable, the top 1000 differential, and the 255 overlap genes are provided in Table S2. Ten interesting examples of genes in these lists are provided in Table 1.

**TABLE 1:** Ten examples of genes with prominent aging connections in the top 1000 differential and/or top 1000 variable gene lists associated with Figure 2

List(s)	Gene name	Protein name	Prominent aging connection
Top 1000 Differential	ADAMTS5	A disintegrin and metalloproteinase with thrombospondin motifs 5	In a mouse model of osteoarthritis, removing the catalytic domain of *Adamts5* prevents cartilage degradation (Glasson et al., 2005)
Top 1000 Variable	APOE	Apolipoprotein E	A large meta‐analysis confirmed that genetic variants of *APOE* are associated with human longevity (Deelen et al., 2019)
Top 1000 Differential	B2M	Beta‐2 microglobulin	Exogenously injecting B2M into mice impairs neurogenesis and cognitive function (Smith et al., 2015)
Top 1000 Variable Top 1000 Differential	COL1A1	Collagen alpha‐1(I) chain	Mice harboring a targeted mutation in *Col1a1* exhibit a shorter life span, hypertension, and reduced bone mineral density (Vafaie et al., 2014)
Top 1000 Variable Top 1000 Differential	FN1	Fibronectin	Mice lacking the EDA exon of *Fn1* have a shorter life span and exhibit irregular skin wound healing (Muro et al., 2003)
Top 1000 Variable	GDF15	Growth/differentiation factor 15	Mice overexpressing human *GDF15* live longer, weigh less, and exhibit enhanced insulin sensitivity (Wang et al., 2014)
Top 1000 Differential	GHR	Growth hormone receptor	Weight is reduced and life span is extended by knocking out *Ghr* in mice (Coschigano et al., 2003)
Top 1000 Variable	IGFBP2	Insulin‐like growth factor‐binding protein 2	Adenovirus‐mediated overexpression of *Igfbp2* reverses diabetes in various mouse models (Hedbacker et al., 2010)
Top 1000 Variable Top 1000 Differential	LEP	Leptin	Mutations in *Lep* cause obesity and type 2 diabetes in mice (Y. Zhang et al., 1994)
Top 1000 Variable Top 1000 Differential	PCK1	Phosphoenolpyruvate carboxykinase, cytosolic [GTP]	Mice overexpressing *Pck1* in skeletal muscle run faster, weigh less, and live longer (Hakimi et al., 2007)

In addition to the top variable and top differential genes, we also directly tested for differentially expressed genes (log_2_ fold >1, FDR <0.05) between healthy adults and healthy old adults (Figure S4a,b). The top over‐represented GO biological process terms varied with the directionality of change. Genes up in healthy older adults tended to overlap significantly with sensory perception and neuronal terms (Figure S4a), while genes down in the healthy older adults showed enrichment with immune and inflammatory response pathways (Figure S4b). However, comparing unhealthy adults to unhealthy older adults revealed different biological terms. While genes up in unhealthy older adults tended to also overlap with neuronal and sensory perception terms (Figure S4c), genes down in unhealthy adults had specific developmental or organ processes significantly enriched but did not show a strong immune or inflammatory signature (Figure S4d). Breaking down the comparison by sex largely revealed a similar set of biological processes significantly overlapped with genes going up or down with age (Figure S4e–h). Interestingly, biological terms that overlapped significantly with genes going up in men were very similar to those overlapping with genes going up in women, with a few differences related to neuronal terms (Figure S4e,g). Both men and women also had similar immune‐related GO terms over‐represented among genes decreasing with age. An exception to this is that response to interferon‐gamma was the top term in men but was not among the top 20 terms in women (Figure S4f,h). Complete lists of the differentially expressed genes between adults and older adults are provided in Table S3.

### 2.3 Machine learning models reveal biological signatures of aging

While differential expression analyses can identify sets of genes that change with age, an orthogonal problem is to use genes to predict human age. This is a classical problem in supervised machine learning called regression analysis, which uses statistical models to estimate a value (i.e., age) based on measured features (i.e., gene expression values from RNA‐Seq). A model is trained to minimize the error between the predicted and known chronological ages, and a cross‐validation strategy is used during training to minimize overfitting. To train a regression model for predicting aging from gene expression, we tried a number of models with implicit feature selection (Figure S5a). Based on accuracy metrics tested across a range of gene sets (Figure S5b, Table S4), random forest (rf) was selected for model fitting. Rf machine learning offers many unique advantages compared with other models, such as having a high predictive power, assigning a relative importance to different inputs, being non‐parametric, and having the capacity to automatically detect non‐linear relationships (Couronne et al., 2018; Touw et al., 2013). Rf was also recently used to generate accurate clocks in mice that can predict either age or life expectancy (Schultz et al., 2020).

**FIGURE 3:**
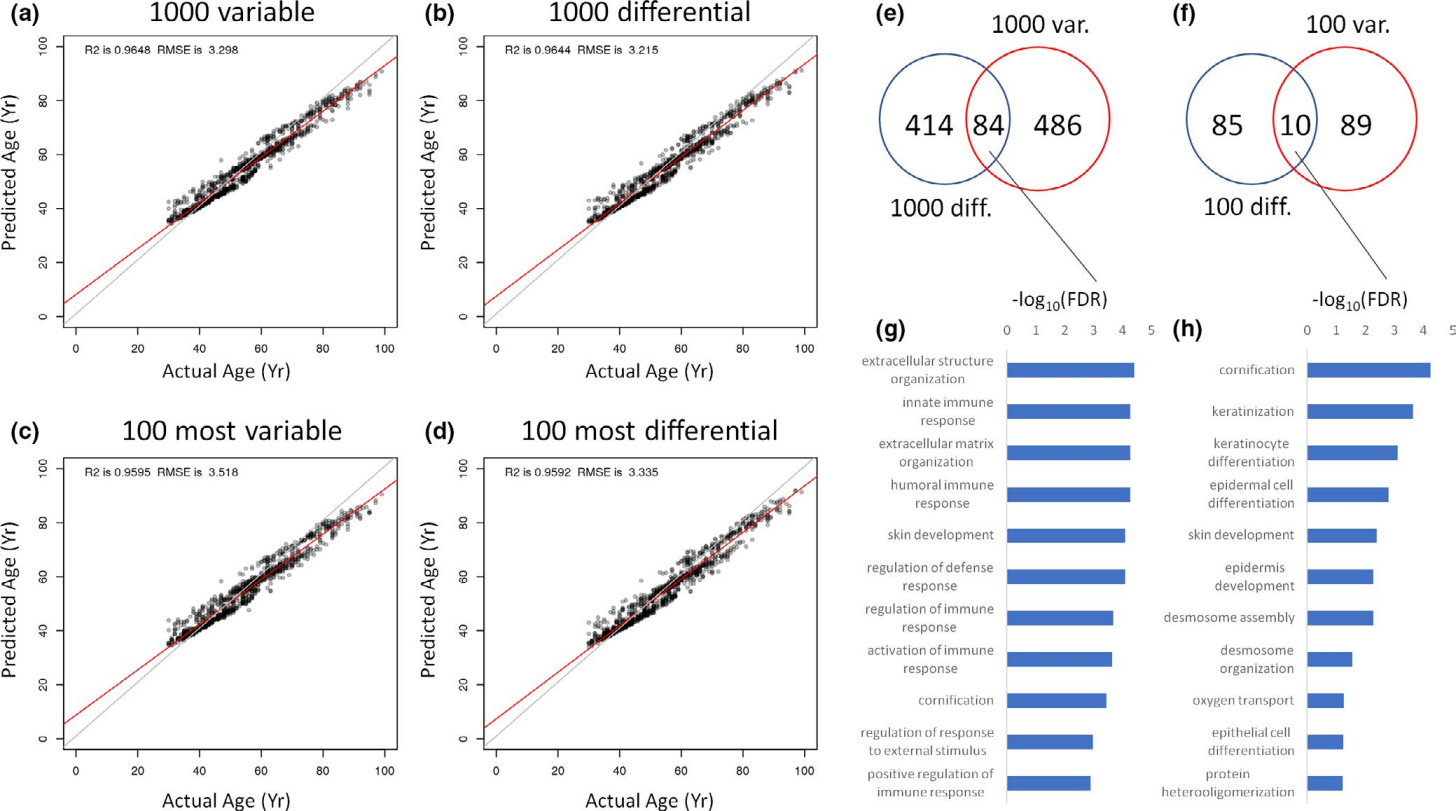
Evaluating the ability of genes to predict human age. (a–d) 10‐fold cross‐validation model of healthy adults based on the top 1000 variable genes (a), top 1000 differential genes (b), top 100 variable genes (c), and top 100 differential genes (d). (e) Overlap in important genes between the top 1000 variable and top 1000 differential models. (f) Overlap in important genes between the top 100 variable and top 100 differential models. (g) Network topology enrichment analysis for the predictive genes common to both the 1000 variable and 1000 differential aging clocks. (h) Network topology enrichment analyses for the predictive genes common to both the 100 variable and 100 differential aging clocks. For both (g) and (h), the 10‐most enriched terms are shown. All models were constructed using random forest machine learning. RMSE, root‐mean‐square error

Next, we evaluated the ability of the model to fit the data using four separate sets of input genes: 1000 most variable genes for healthy individuals (Figure 3a), 1000 most differential genes between healthy adults and healthy older adults (Figure 3b), 100 most variable genes across all healthy adults (Figure 3c), and the 100 most differential genes between healthy adults and healthy older adults (Figure 3d). All four models were able to accurately predict the ages of healthy adults (Figure 3a–d) with *R* ^2^ values above 0.95 and root‐mean‐square error (RMSE) values lower than 4 years. Since the process of training the importance of each gene can be estimated (see Methods), each model ended up with a unique set of important genes, which were defined as having an importance score >1 (Figure 3e). Only 84 genes were commonly important in both the model trained on the top 1000 variable genes and the model trained on the top 1000 differential genes (Figure 3e). When gene overlap was compared between the top 100 variable and differential models, only 10 genes were commonly important (Figure 3f). Only five genes—*HBB*, *KRT1*, *KRT13*, *KRT14*, and *KRT16*—were important in all four models. Network‐based enrichment analysis of the 84 important genes common to the 1000 variable and 1000 differential lists shows that extracellular matrix organization, immune responses, and cornification were among those significantly enriched (Figure 3g). For the 10 genes commonly important to the 100 variable and 100 differential lists, enrichment themes included cornification, keratinization, and skin development (Figure 3h). The list of important genes for each model presented in Figure 3 is provided in Table S5. The top 10 important genes for the top 1000 variable and top 1000 differential aging clocks are highlighted in Table 2.

**TABLE 2:** The top 10 predictive genes are shown for the top 1000 variable and top 1000 differential aging clocks presented in Figure 3. For each gene, its quantified importance in the random forest prediction model is provided

Aging clock	Gene name	Protein name	Importance
1000 Variable	KRT6C	Keratin, type II cytoskeletal 6C	14.78989842
1000 Variable	KLK6	Kallikrein‐6	14.13181626
1000 Variable	SPRR1B	Cornifin‐B	12.23451207
1000 Variable	MMP10	Stromelysin‐2	10.47366716
1000 Variable	CALML3	Calmodulin‐like protein 3	9.350379696
1000 Variable	CEACAM5	Carcinoembryonic antigen‐related cell adhesion molecule 5	8.906136426
1000 Variable	GRP	Gastrin‐releasing peptide	8.344751464
1000 Variable	FGA	Fibrinogen alpha chain	7.586499443
1000 Variable	KRT6B	Keratin, type II cytoskeletal 6B	7.481560771
1000 Variable	PRSS2	Trypsin‐2	7.316476098
1000 Differential	REG3A	Regenerating islet‐derived protein 3‐alpha	40.34504236
1000 Differential	NR2E1	Nuclear receptor subfamily 2 group E member 1	13.02751594
1000 Differential	DEFB119	Beta‐defensin 119	12.62814497
1000 Differential	MIR10B	N/A	11.9933185
1000 Differential	MIR100	N/A	10.6716294
1000 Differential	KRT6C	Keratin, type II cytoskeletal 6C	10.30831422
1000 Differential	DPT	Dermatopontin	10.11309756
1000 Differential	HLA‐F	HLA class I histocompatibility antigen, alpha chain F	9.340543895
1000 Differential	KCNC2	Potassium voltage‐gated channel subfamily C member 2	8.923200977
1000 Differential	INS	Insulin	8.647671153

For a comparison, we assessed which important genes were prioritized by a cubist machine learning model compared with the Rf machine learning model (Figure S6). Cubist and Rf models using either top variable (Figure S6a) or top differential (Figure S6b) genes uniquely prioritized different sets of inputs. For the variable gene models, 357 important genes were unique to the Rf model, 117 important genes were unique to the cubist model, and 213 important genes were common to both models (Figure S6a). For the differential gene models, 303 important genes were unique to the Rf model, 161 important genes were unique to the cubist model, and 195 important genes were unique to both models (Figure S6b). The genes uniquely prioritized by each model implicated a different set of biological processes (Figure S6c–f). Lists of important genes associated with these Rf and cubist models are provided in Table S6.

### 2.4 Transcriptomic age predictors are affected by health status

**FIGURE 4:**
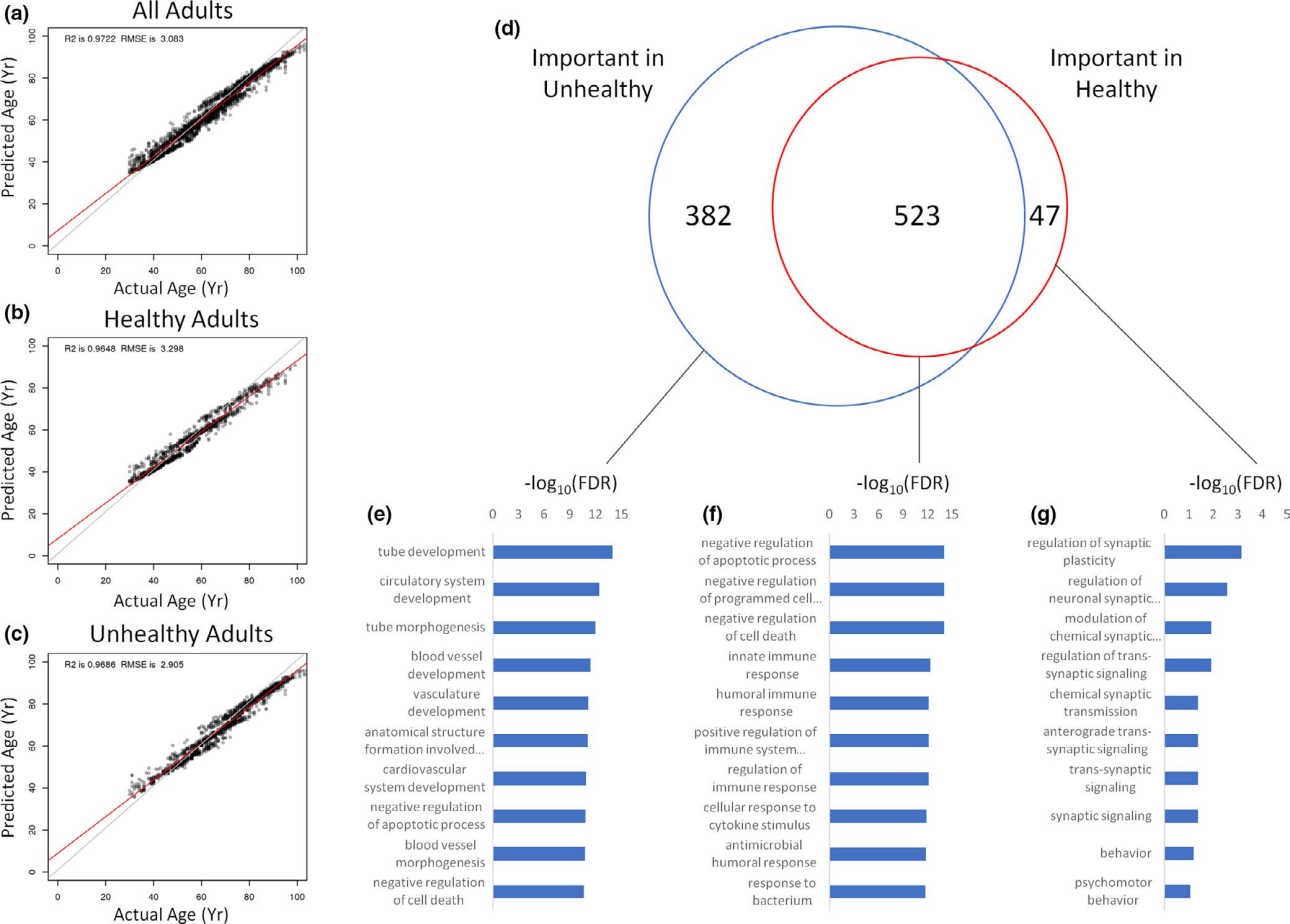
Health status affects aging clocks. (a) Model fit on all adults used to predict all adults, regardless of health status. (b) Model fit on healthy adults used to predict healthy adults. (c) Model fit on non‐healthy adults used to predict non‐healthy adults. (d) Overlap of important genes from each model. (e) Network topology‐based analysis of predictive genes in the non‐healthy model but not in the healthy model. (f) Network topology‐based analysis of predictive genes common to both healthy and unhealthy models. (g) Network topology‐based analysis of important genes predictive in the healthy model but not in the unhealthy model. For each enrichment analysis (e–g), the 10‐most enriched terms are shown. All models were constructed using random forest machine learning. RMSE, root‐mean‐square error

Since all studies included reported on the health status of individuals, we next tested whether models trained on unhealthy (all except healthy) adults or both healthy and unhealthy adults could predict age and whether different genes would be important in these different models compared to a model trained on just the healthy adults. We observed that Rf machine learning could accurately model age in all adults (healthy + unhealthy; Figure 4a), just healthy adults (Figure 4b), and just unhealthy adults (Figure 4c). We then tested the overlap between important genes among these models (Figure 4d). There were 523 important genes common to both the healthy and unhealthy models, suggestive of an overlapping RNA aging signature between healthy and unhealthy individuals. Performing a network topology‐based enrichment analysis on the 382 genes uniquely important for the unhealthy model largely identified vascular terms (Figure 4e). The 523 common genes highlighted cell death and various immune response terms as being significantly overlapped (Figure 4f). The 47 genes uniquely important in the healthy model of aging revealed regulation of neuroplasticity and other neuronal terms to be significantly enriched (Figure 4g). Table S7 lists all of the important genes prioritized by each aging clock shown in Figure 4.

### 2.5 Sex‐specific models exhibit disparate aging signatures

Analogously to health status‐specific aging, we hypothesized that we could develop sex‐specific models of aging and that there would be differences in the genes that are important for prediction in these models. Indeed, we developed predictive models that were fit on healthy males and females (Figure S7a), healthy males (Figure S7b), and healthy females (Figure S7c). By comparing the overlap of important genes (Figure S7d), we find 440 genes that are unique to healthy females, 338 common genes that are important in both healthy males and healthy females, and 65 genes that are unique to healthy males. Uniquely important genes in healthy females are enriched for a variety of terms, including developmental processes, cytokine‐mediated signaling, and negative regulation of cell death (Figure S7e). Common important genes are enriched for MAPK signaling, innate immune response, ERK signaling, wounding response, and other processes (Figure S7f). The model trained on healthy males had just 65 uniquely important genes that were enriched for regulation of ion transport and immune processes (Figure S7g). Lists of the important genes prioritized by each sex‐specific or sex‐independent model shown in Figure S7 are provided in Table S8.

### 2.6 Aging signatures are tissue‐dependent

**FIGURE 5:**
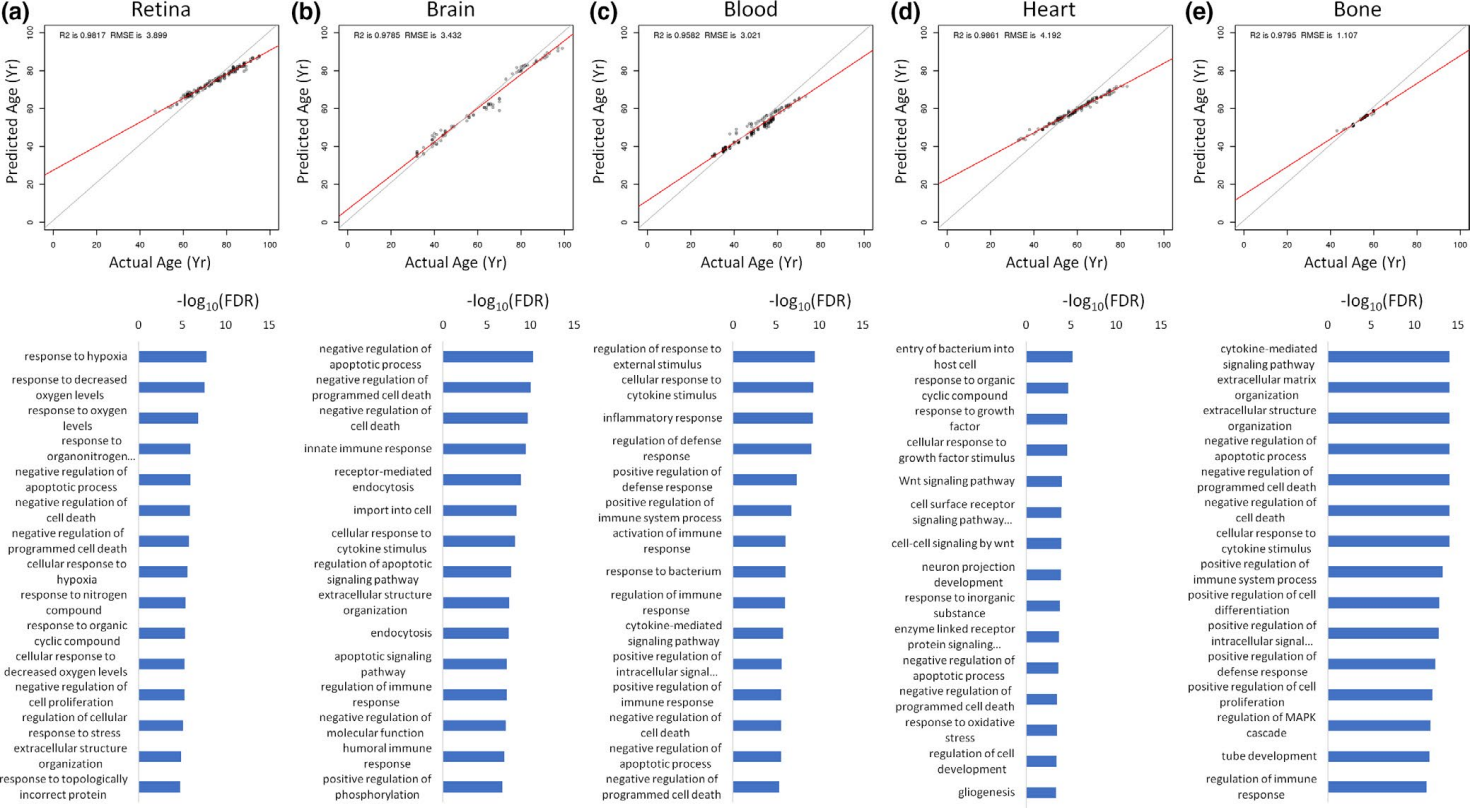
Tissue‐specific aging models. For retina (a), brain (b), blood (c), heart (d), and bone (e) tissues, predictive aging clocks were trained on healthy samples using the top 1000 variable genes. A network‐based enrichment analysis was performed on predictive genes for each tissue. The 15 most enriched terms are shown. All models were constructed using random forest machine learning. RMSE, root‐mean‐square error

Since our dataset represents a large collection of transcriptomes from diverse tissues (Figure 1b), we wondered if we could develop tissue‐specific aging signatures with our machine learning approach. To do this, we trained tissue‐specific models for the following five tissues that had the highest number of healthy samples: retina (Figure 5a), brain (Figure 5b), blood (Figure 5c), heart (Figure 5d), and bone (Figure 5e). Our clocks were able to accurately model age within each tissue, with *R* ^2^ values ranging from 0.96 to 0.99 and RMSE values ranging from 1.11 to 4.19 years (Figure 5). For each tissue model we trained, we again identified genes important for model accuracy and used a network topology‐based GO enrichment analysis to identify terms specific to predictive aging genes in each tissue. Response to hypoxia, negative regulation of cell death, regulation of cell proliferation, regulation of stress response, and extracellular matrix organization was enriched in the retina‐specific aging model (Figure 5a). For the brain, negative regulation of cell death, immune response, endocytosis, and extracellular structure organization terms were among the most enriched (Figure 5b). A blood‐specific aging model identified genes enriched for inflammatory response, activation of the immune system, and negative regulation of cell death (Figure 5c). In the heart, important genes were associated predominantly with entry of bacterium into host cell, response to growth factor, Wnt signaling, development, and negative regulation of cell death (Figure 5d). Finally, a bone‐specific model identified genes predictive of aging that were significantly enriched for extracellular matrix organization, negative regulation of cell death, immune response, and regulation of MAPK (Figure 5e).

The complete list of genes prioritized for each clock is provided in Table S9. A total of six predictive genes (*CHI3L2*, *CIDEC*, *FCGR3A*, *RPS4Y1*, *SLC11A1*, and *VTCN1*) were shared by all five of these tissues, while 43 were shared by four or more tissues (Table S9). We additionally compared our tissue‐specific important genes to the top 20 genes previously used for age prediction in human skeletal muscle (Mamoshina et al., 2018) and a set of 54 genes previously used to predict age in human blood (Hannum et al., 2013). Compared with the 20 human skeletal muscle genes previously utilized by Mamoshina et al. (2018), the genes *GREM1* and *VSNL1* were important in at least one of our tissue‐specific clocks (Table S9). Compared with the 54 human blood genes previously used by Hannum et al. (2013), the genes *AK5*, *NEFH*, *NT5E*, and *RGMA* were important in at least one of our tissue‐specific clocks (Table S9).

### 2.7 Model accuracy is impacted by the human cohort being tested

**FIGURE 6:**
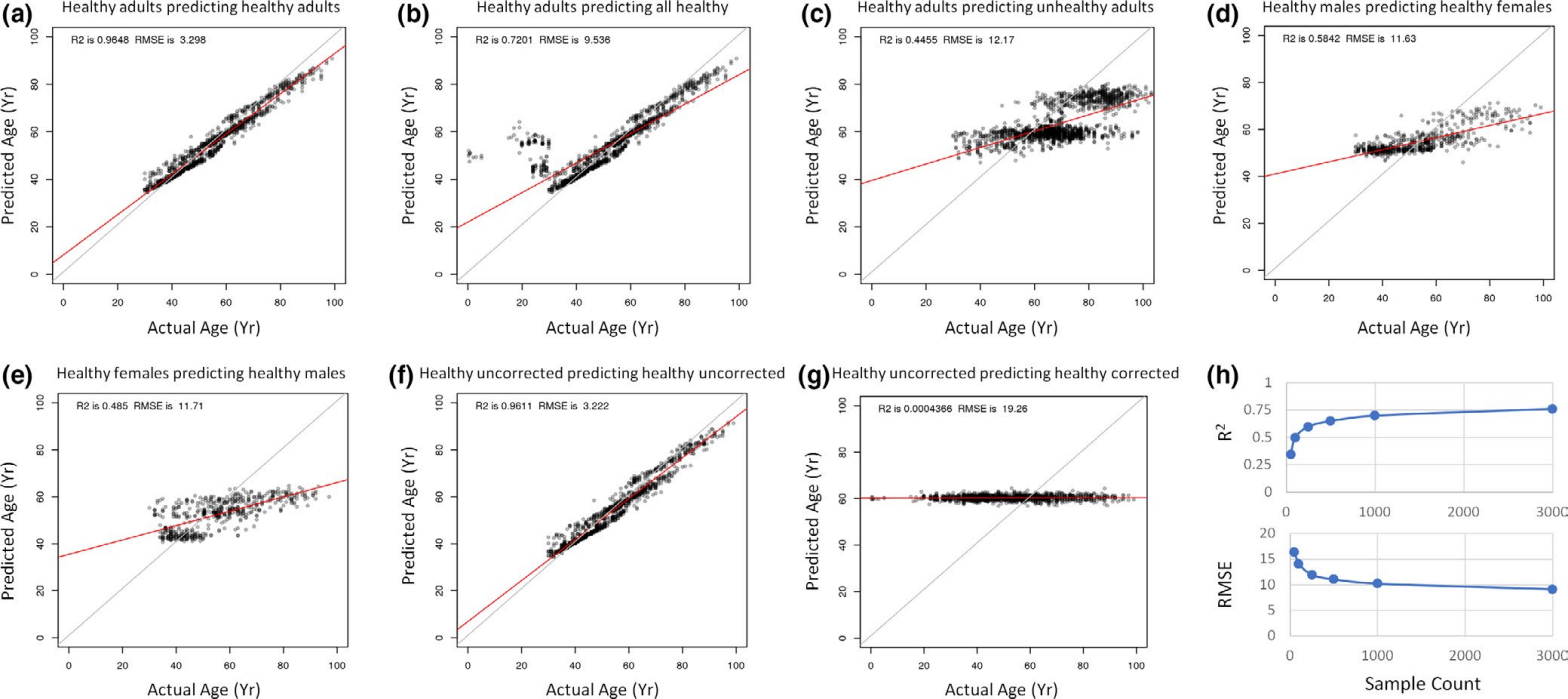
Evaluating the generalizability of predictive aging models. (a) 10‐fold cross‐validated model of healthy adults predicting age of healthy adults. (b) 10‐fold cross‐validated healthy adult model predicting age of all healthy individuals, including younger individuals. (c) 10‐fold cross‐validated model of healthy adults used to predict unhealthy adults. (d) 10‐fold cross‐validated healthy adult male model predicting age of all healthy females. (e) 10‐fold cross‐validated healthy adult female model predicting age of all healthy males. (f) 10‐fold cross‐validated model of healthy adults predicting on data without batch correction. (g) 10‐fold cross‐validated model of healthy adults trained on data without batch correction used to predict batch‐corrected data. (h) Model error as a function of sample size for all individuals. As sample size increases, the *R* ^2^ value increases and the RMSE value decreases. All models were constructed using random forest machine learning. RMSE, root‐mean‐square error

We next went on to explore how these models generalized to other datasets. While a model trained on healthy adults is able to accurately predict healthy adult ages (Figure 6a), we found that the same model has trouble predicting the ages of younger (<30 years) individuals (Figure 6b). A model trained on healthy adults also fared relatively poorly when it was used to predict the age of unhealthy adults (Figure 6c). Sex‐specific models similarly displayed an impaired predictive performance when tasked with predicting age in samples from the opposite sex (Figure 6d,e). Importantly, batch‐effect correction completely changed which genes were estimated to be important for model accuracy (Table S10). While an uncorrected model could be trained to accurately predict age (Figure 6f), the model relied heavily on almost all 1000 variable genes (Figure S2i). Genes that were predictive specifically in the batch‐corrected model included general biological terms such as negative regulation of cell death, extracellular structure organization, humoral immune response, innate immune response, and ERBB signaling (Figure S2j). Conversely, the predictive genes specific for the uncorrected model tended to involve tissue‐specific terms such as sensory perception and neuron projection development (Figure S2l). A common set of 194 genes important in both the batch‐corrected and uncorrected models was enriched for immune responses, aging, homeostasis, and exocytosis (Figure S2K). Importantly, models based on uncorrected expression values completely failed to predict age from corrected datasets (Figure 6g). Finally, to test how sample size affected model accuracy, we repeatedly sub‐sampled our entire dataset prior to training and observed that models based on just 50 random samples had an average *R* ^2^ of 0.35 and an average RMSE of 16.43 years (Figure 6h, Table S11). However, by progressively increasing the number of samples, we were able to reach an *R* ^2^ of 0.76 and an average RMSE of 9.12 years (Figure 6h, Table S11). Interestingly, a model trained on just the top 100 variable genes can still achieve an *R* ^2^ of 0.72 and an RMSE of 9.69 years (Table S11). Similarly, a model trained on just the top 100 differential genes achieves an *R* ^2^ of 0.72 and an RMSE of 9.65 years (Table S11).

### 2.8 An online, interactive tool for predicting human age

While our models are useful tools for identifying genes that are relevant to aging, they can also be used to directly make age predictions in a given RNA‐Seq dataset. Therefore, to help scientists interested in predicting the age of their samples without the need for computational resources and bioinformatics expertise, we have developed a simple Bioinformatics Utility for RevealiNg Senescence (BURNS). The link to this tool is as follows: http://burns.salk.edu/. This tool reads in an uploaded raw count table and outputs the predicted age from a user‐specified machine learning model. The two model options to choose from utilize either the top 100 variable genes or the top 100 differential genes. The tool attempts to combine the user‐provided data with our large dataset as a new batch, performs normalization and batch‐effect correction, and then uses the specified model to predict the age of each sample.

## 3 DISCUSSION

This manuscript presents a multitude of highly accurate and novel transcriptomic aging clocks representing different tissues, sexes, and human cohorts (Table S12). For multiple different datasets, we were able to accurately predict age by using genes that vary their expression with age or are differentially expressed between older adults and younger adults. Our data therefore suggest that genes which change their expression level with age make for excellent age predictors. Moreover, many of the genes present in the top 1000 variable or top 1000 differential lists are potent regulators of aging or age‐related disease. For example, an analysis of our entire dataset identified *APOE* among the top 1000 variable genes. Genetic variants of *APOE* are unambiguously associated with human longevity (Deelen et al., 2019) and Alzheimer's disease (Kunkle et al., 2019). In addition, mice lacking *Apoe* are atherosclerosis‐prone (Zhang et al., 1992). Another example is *PCK1*, which was present in both the top 1000 variable and top 1000 differential lists. Overexpressing *Pck1* in mice extends life span and enhances exercise capacity (Hakimi et al., 2007). We recently found that many proteins which change their expression level with age in plasma can accurately predict human age and are also direct regulators of life span and/or age‐related disease (Lehallier et al., 2020). Thus, this attribute appears to be shared between genes and proteins.

Another interesting finding was that each tissue‐specific aging clock uniquely prioritized different genes for age prediction. In turn, these unique lists of important genes were associated with different biological processes. The gene with the highest importance in the retina, brain, blood, heart, and bone aging clocks was *FNDC1*, *SPINK1*, *XIST*, *RYR3*, and *CYP1B1*, respectively. The most enriched terms were response to hypoxia, negative regulation of apoptotic process, regulation of response to external stimulus, entry of bacterium into host cell, and cytokine‐mediated signaling pathway in the retina, brain, blood, heart, and bone age predictors, respectively. While each gene list and set of enriched terms was unique to each clock, there were interesting overlaps. The six genes *CHI3L2*, *CIDEC*, *FCGR3A*, *RPS4Y1*, *SLC11A1*, and *VTCN1* were important in all five tissue‐specific clocks. Each of the five tissue clocks had multiple different terms pertinent to cell death that were significantly enriched. Immune processes were also enriched in the brain (i.e., innate immune response, cellular response to cytokine stimulus, regulation of immune response, and humoral immune response), blood (i.e., cellular response to cytokine stimulus, inflammatory response, regulation of defense response, positive regulation of defense response, positive regulation of immune system process, activation of immune response, response to bacterium, regulation of immune response, cytokine‐mediated signaling pathway, and positive regulation of immune response), heart (i.e., entry of bacterium into host cell), and bone (i.e., cytokine‐mediated signaling pathway, cellular response to cytokine stimulus, positive regulation of immune system process, positive regulation of defense response, and regulation of immune response) aging clocks. The term extracellular structure organization was also enriched in the retina, brain, and bone age predictors. A recent study in mice by Schaum et al. (2020) similarly found that immune system and extracellular matrix processes were altered with age across multiple different tissues. Unique changes in immune system genes have also been identified in exceptionally long‐lived animals, such as naked mole rats and bowhead whales (Johnson et al., 2019).

Looking at the similarities and differences between genes and terms implicated by different tissues, our interpretation is that a larger proportion of aging changes are tissue‐specific while a smaller proportion of aging changes commonly span multiple different tissues. Analogous results were obtained by Shavlakadze et al. (2019) in a rat transcriptomic aging study. The authors found that, although gene trajectories with age were largely organ‐specific, a small portion of gene changes occurred in multiple different tissues. These data have important implications for anti‐aging interventions intending to improve human health span. Specifically, some anti‐aging interventions may need to be tissue‐specific to safely promote health span extension. Indeed, work in animal models has shown that anti‐aging interventions can mediate significantly disparate effects when applied in different tissues (Smith et al., 2020). However, it is also possible that a small number of global interventions may be capable of safely increasing human health span.

Although our tissue‐specific aging clocks heavily implicated inflammatory terms, one surprising discovery was that multiple different immune system terms were enriched among genes downregulated with age in our larger cohort of healthy adults, which contained data from all healthy tissues. This strong inflammatory theme was not observed in genes upregulated with age in our healthy adults. This stands out in contrast to transcriptomic aging studies performed by others (de Magalhaes et al., 2009; Zeng et al., 2020), which reported that inflammatory genes are upregulated with age. Collectively, our study and these other studies may be highlighting the fact that some aspects of the immune system become hyperactive while others become less capable with age. For example, chronic inflammation increases with age while the ability to ward off active infections is decreased in the elderly (Furman et al., 2019). Therefore, the results from our healthy cohort may be highlighting processes that become suppressed with normal aging. For example, the second‐most and fourth‐most enriched results among downregulated genes in healthy adults were adaptive immune response and humoral immune response, respectively. These enriched terms correspond with our understanding of why high‐dose influenza vaccines are recommended for individuals aged 65 years or older (Lee et al., 2018). Another interpretation is that the genes implicating some of these enrichment terms may be suppressors of the immune system. Thus, their decline with age may actually indicate increased inflammation. A closer, functional exploration of the relevant genes is required to better understand the directionality of these immune system processes. An alternative possibility is that these immune enrichment results may reflect the unique healthy tissue distribution of our transcriptomic meta‐analysis.

Our findings also highlight the importance of either training a model to be generalized or cohort‐specific. For example, a clock trained on all healthy adults (aged 30+ years) has a *R* ^2^ of 0.96 and a RMSE of 3.3 years. However, when this same clock is tasked with predicting age in a cohort of all healthy individuals (including those aged <30 years), the *R* ^2^ drops to 0.72 and the RMSE increases to 9.54 years. Similarly, a clock trained on healthy adults has a lower accuracy when applied to a group of unhealthy adults (*R* ^2^ = 0.45, RMSE = 12.17 years). To further explore the generalizability of these clocks, we repeatedly sub‐sampled our dataset and predicted on the rest. We found that there was a strong correlation between the number of transcriptomic samples and the overall accuracy of the model. When 3000 samples were included, we created a more generalized clock with a *R* ^2^ value of 0.76 and a RMSE of 9.12 years. Generalized transcriptomic aging clocks with substantially increased accuracy will likely require a much larger number of high‐quality samples (i.e., >10,000). Ultimately, the desired application of an aging clock will dictate how specific or generalized a model should be. One of the proposed applications of aging clocks is to accelerate anti‐aging clinical trials by assessing whether or not biological age is decreased in response to a given intervention (Fahy et al., 2019). For this application, biological age would be measured in the same cohort prior to the clinical trial and at the end of the clinical trial. Here, it would make sense to generate a novel, cohort‐specific aging clock. However, other applications of aging clocks include assessing whether or not the delta between predicted age and chronological age is reflective of patient health (Horvath & Raj, 2018). For this, a generalized clock will be more useful.

All of the transcriptomic samples used in this study contained patient health information. Each RNA‐Seq dataset was associated with a disease condition or a healthy/non‐diseased state. If a sample was derived from a patient with a diagnosed disease, we labeled the sample as “unhealthy.” If it was derived from a healthy/non‐diseased control patient, it was labeled as “healthy.” The genes prioritized by our aging clock models differed considerably between our “healthy” cohort and our “unhealthy” cohort. Namely, 47 important genes were unique to the “healthy” clock and 382 important genes were unique to the “unhealthy clock.” This corroborates a recent study which reported a “healthy” transcriptomic aging signature that was distinct from disease‐associated gene changes (Zeng et al., 2020). However, 523 of the same important genes in our study were utilized by both age prediction models. An enrichment analysis of these common genes strongly implicated the regulation of cell death and different immune system responses.

A closer look at the enrichment terms between “healthy” and “unhealthy” aging unveils some intriguing differences and similarities. For genes that increase with age, the overlap was substantial. Indeed, the top four terms that appeared in the same descending order of significance are as follows: photoreceptor cell differentiation, sensory perception of light stimulus, detection of external stimulus, and detection of abiotic stimulus. Seventeen of the top 20 enriched terms associated with genes that increase with age were shared between the “healthy” and “unhealthy” cohorts. In the “unhealthy” group, the disparate terms were cognition, regulation of ion transmembrane transport, and signal release. In the “healthy group,” the disparate terms were sensory organ morphogenesis, locomotory behavior, and axon development. In contrast, the results for genes downregulated with age were entirely different between the “healthy” and “unhealthy” datasets. While 16 of the enriched terms were related to the immune system in the “healthy” group, only one inflammatory term—response to tumor necrosis factor—was identified in the “unhealthy” group. The remaining four terms in the “healthy” cohort were extracellular structure organization, positive regulation of cell activation, regulation of cell‐cell adhesion, and positive regulation of cell adhesion. The terms implicated by genes downregulated with age in “unhealthy” individuals were much more diverse, spanning themes such as actin filaments, muscle, heart, and vasculature. Further efforts are warranted to better understand the transcriptomic aging signatures that define “healthy” vs. “unhealthy” aging.

Given the growing interest in aging clocks, including commercial patenting, it is useful to discuss their practical utility in more detail. Because aging clocks appear to routinely capture biological age—which correlates with various health parameters and outcomes—they have the potential to significantly accelerate anti‐aging intervention testing in animal models and anti‐aging clinical trials in humans. For example, the laboratory of David Sinclair recently used a machine learning model trained on frailty index components to predict the efficacy of anti‐aging interventions up to a year in advance in mice (Schultz et al., 2020). In humans, a small, exploratory study from the laboratory of Steve Horvath suggests that treatment with various pharmaceuticals, including metformin, can reverse biological age (Fahy et al., 2019). Since life span studies in humans are not practical, aging clocks offer a highly appealing mechanism to assess the ability of various interventions to elongate life span and/or health span. The development of tissue‐specific clocks may also be useful for assessing the ability of a therapy to mitigate disease or age‐related decay unique to a particular tissue. For example, an epigenetic aging clock was recently made for skeletal muscle (Voisin et al., 2020). Such a clock could be used to screen for therapies that rejuvenate muscle or protect against sarcopenia. More broadly, machine learning could be used for other applications in biogerontology, such as diagnostics for age‐related disease. Indeed, deep learning models have already been developed for the detection of Alzheimer's disease (Jo et al., 2019). As we have done in this paper, it is also clear that the features (e.g., genes, proteins, methylated DNA status) chosen by these machine learning models can teach us more about aging if subsequently annotated using functional databases. One advantage of clocks comprised of RNA or protein is that they are conducive to straightforward enrichment analyses.

In summary, we have utilized machine learning models and expression analyses to gain novel insights into the transcriptomics of human aging. In addition to generating novel multi‐tissue and tissue‐specific aging clocks, we create and freely share the processed dataset and provide an online age prediction tool named Bioinformatics Utility for RevealiNg Senescence (BURNS), which includes batch‐effect correction and normalization to generate models trained across all 3060 transcriptomic samples. This maximizes the generalizability of the tool. Future research efforts should aim to better understand the composition of these age prediction models and determine whether or not pharmaceutical targeting of key human aging genes (i.e., those that significantly change with age or are important for age prediction) can promote healthier aging. Ultimately, the theoretical ability of a high‐quality aging clock to accelerate anti‐aging clinical trials needs to be explicitly tested in a large patient cohort.

## 4 EXPERIMENTAL PROCEDURES

### 4.1 Data curation and filtration

We manually curated a large list of publicly available human bulk RNA‐Seq datasets from the Sequence Read Archive (Leinonen et al., 2011) which included information on the age of the samples. We did not consider datasets which only focused on children, since we did not want to further complicate the aging signals with developmental signals.

For each RNA‐Seq run, we downloaded the first 12 million reads. For paired‐end samples, we chose to exclude the second read for consistency between single‐ and paired‐end sequenced samples. We then mapped and quantified the reads with STAR (Dobin et al., 2013) using the [options]. We chose the hg38 RefSeq annotation for quantifying genes. The resulting raw gene expression counts were collated into a data table that was imported into the R 3.5 (R Foundation for Statistical Computing, Vienna, Austria) computational environment for preprocessing and modeling. We next imposed three main filters. First, samples that had fewer than two million reads mapping to exons were excluded to avoid gene sampling bias based on the distribution of read counts seen across all samples. We further removed batches that we identified to decrease the accuracy of downstream predictions. The procedure for this is described below. Finally, for the remaining 3060 samples, we removed genes with average raw expression below 50, leaving 13,388 highly expressed genes for downstream processing. These remaining 13,388 genes were subsequently analyzed to generate transcriptomic aging clocks and to perform differential expression analyses.

### 4.2 Normalization and batch‐effect correction

We applied a TMM normalization from the edgeR package (Robinson et al., 2010) which corrects for sequencing depth and sequencing library composition. We further added a pseudo‐count and log2‐transformed the normalized counts to normalize the importance of genes. Since we observed large batch effects when visualizing the first two principal components, combat batch effect correction from the sva package v3.32.1 (Leek et al., 2012) was used to correct for batch effects across studies starting with the log2‐transformed TMM normalized counts. The top 10, 100, and 1000 most variable corrected genes were then saved for machine learning training. The top 1000 most variable genes were clustered and a manual cutoff of 30 and 70 years was determined to delineate adults and old adults, respectively, based on the clustering of these genes across all healthy samples ordered by their age. A cutoff of 30 years for adults was assumed to minimize any possible developmental effects confounding the aging signature while a threshold of 70 years ensured a large sampling of 965 older adults.

### 4.3 Differential expression

We used the edgeR v3.26.7 (Robinson et al., 2010) for differential expression analyses. We compared adults (30–69 years) to older adults (70+ years) and included the batch as a blocking variable in the design, using the gene‐wise negative binomial generalized linear models with quasi‐likelihood tests. The top 1000 and 1000 differential genes between healthy adults and healthy aged adults were selected for machine learning, starting from batch‐corrected normalized counts. We also tested differential genes between unhealthy adults and older adults. This was performed separately for males and females.

### 4.4 Machine learning

The Caret R package v6.0.84 (Kuhn, 2008) was used for machine learning. Models were trained using a repeated 10‐fold cross‐validation to minimize overfitting and using a tune length of 5 (5 values were tested for each hyperparameter), and two repeats of the 10‐fold cross‐validation were used for hyperparameter tuning, using a selection criterion that selected the simplest model that was within one standard error of the model with the best RMSE. The one standard error rule was used to further help avoid overfitting. Hyperparameter tuning and cross‐validation was carried out for each model training. Prior to fitting, the corrected normalized data were scaled to reduce bias from higher‐expressed genes. Finally, we limited the possible predictions to 0–110 years which encompassed the age range of our entire dataset. We tested several popular models for age regression training using the 100 and 1000 most variable genes as well as the top 100 and 1000 differential genes for model construction. Specifically, we tested a rule‐based model tree (cubist), generalized linear model via penalized maximum likelihood (glmNet), multivariate adaptive regression splines (gcvEarth), boosted smoothing spline (bstSm), random forest (rf), and elastic net (enet) models because they include implicit feature selection as part of the model training. This is important because a model with 1000 features (i.e., genes) runs the risk of overfitting, essentially memorizing the age of the training samples. In the process of training, these models reduce the number of features and improve the generality of the fits. We selected the random forest model (rf) based on the overall performance (RMSE, *R* ^2^, and mean absolute error) across all four training sets. For the random forest model, the “mtry” parameter, which is defined as the number of variables randomly sampled as candidates at each split, was tuned using values of 2, 9, 44, 211, and 1000 for models with 1000 genes and values of 2, 26, 51, 75, and 100 for models with 100 genes. The optimal mtry parameters were 211 for the model trained on top 1000 variable genes in healthy adults, 1000 for the model trained on top 1000 differential genes in healthy adults, and 26 for the models trained on the top 100 variable or differential genes in healthy adults. For all models, in practice, the model accuracy was insensitive to the mtry hyperparameter value as long as it was at least 10. Model‐specific variable importance was calculated using default variable importance wrappers in the Caret package for “rf” and “cubist” models. Briefly, for random forest, variable importance was determined using an out‐of‐bag strategy. For each tree, the prediction accuracy on the out‐of‐bag portion of the data is recorded. The mean squared error (MSE) is computed on the out‐of‐bag data for each tree, and then, MSE is recomputed after permuting a variable. The differences in MSE are averaged and normalized by the standard error. For the cubist models, the output contains variable usage statistics. It gives the percentage of times where each variable was used in a linear model. The variable importance used is a linear combination of the usage in the rule conditions and the model. To test model accuracy as a function of training sample size, we repeatedly trained (*n* = 9) the model on a subset of the samples and predicted on the remaining samples for sample sizes of 50, 100, 250, 500, 1000, and 3000 on the entire filtered dataset. This nested cross‐validation strategy showed similar model accuracy estimates to the repeated 10‐fold cross‐validation with the one standard error model selection that was used during training. This along with the relative insensitivity of the performance to the hyperparameter was used to justify not performing a full nested cross‐validation for all models.

Importantly, we first only applied the sample raw count filter and the gene expression filter (see “Data curation and filtration” section above). However, we found that removing entire batches could significantly improve the predictive power of our machine learning models. Therefore, we compared the predictive power of our model trained on all batches to models with each batch iteratively removed to estimate the importance of the batch in our combined model. For each batch, we recorded the RMSE, *R* ^2^, and mean absolute error of the model with the missing batch. Only batches which had decreased performance by all three metrics were removed from our study. This resulted in the removal of 27/58 batches, but also an overall dramatic improvement of age predictions in our filtered dataset.

### 4.5 Enrichment analyses and plots

Enrichment analyses were performed similarly to before (Johnson et al., 2020). We used WebGestalt 2019 (Liao et al., 2019) to perform enrichment analyses on sets of genes. We used the network topology‐based enrichment analysis (NTA) with the PPI BioGRID as the network and network expansion with the number of neighbors set to half the size of the input list. We report the top enriched GO biological process (The Gene Ontology Consortium, 2019) terms, filter for terms with less than 1000 genes, and sort by ‐log_10_(FDR), with FDR of zero plotted as 14. We chose to plot the enrichment ratio instead for Figure S4, as most of the top terms had a FDR of zero. Heatmaps were generated with the gplots heatmap.2 function, barplots were generated with Excel or barplot functions in R, and pie charts and boxplots were generated with the R pie and boxplot functions, respectively. PCA plots were generated in R using the prcomp function, and scatter plots were generated with the R plot function using a linear regression for line fitting. Histograms were generated with the R hist function, and all other plots were generated in Excel. The proportional Venn diagrams were generated with BioVenn (Hulsen et al., 2008). We chose to perform GO BP analyses instead of GO Cellular Component and Molecular Function analyses because GO BP terms highlight biological programs that arise from molecular activity.

### 4.6 Interactive age prediction tool

To enable researchers to annotate their RNA‐Seq samples, we developed Bioinformatics Utility for RevealiNg Senescence (BURNS) using the Shiny R environment. This tool can be used to upload your own samples (samples are not stored), perform TMM normalization, apply Combat batch‐effect correction, and then predict the age of samples based on a model trained on the top 100 variable or the top 100 differential genes across all 3060 samples. This online tool is hosted at http://burns.salk.edu free of charge.

## CONFLICT OF INTEREST

The authors have no conflicts of interest to declare.

## AUTHOR CONTRIBUTIONS

MNS performed the experiments and contributed to project design, data collection, and manuscript writing. AAJ contributed to project design, data collection, and manuscript writing.

## Supporting information

Figure S1

Figure S2

Figure S3

Figure S4

Figure S5

Figure S6

Figure S7

Tables S1 and S12

Supplementary Material

## Data Availability

All of the RNA‐Seq datasets analyzed in this study are publicly available in the Sequence Read Archive (https://www.ncbi.nlm.nih.gov/sra). The raw gene count table and metadata are available from Mendeley (http://dx.doi.org/10.17632/92rgnswtn8.1). We additionally created a publicly available, online interactive tool for using two of our aging clock models: http://burns.salk.edu.

## References

[acel13280-bib-0001] Benayoun, B. A. , Pollina, E. A. , Singh, P. P. , Mahmoudi, S. , Harel, I. , Casey, K. M. , Dulken, B. W. , Kundaje, A. , & Brunet, A. (2019). Remodeling of epigenome and transcriptome landscapes with aging in mice reveals widespread induction of inflammatory responses. Genome Research, 29(4), 697–709. 10.1101/gr.240093.118 30858345 PMC6442391

[acel13280-bib-0002] Bocklandt, S. , Lin, W. , Sehl, M. E. , Sanchez, F. J. , Sinsheimer, J. S. , Horvath, S. , & Vilain, E. (2011). Epigenetic predictor of age. PLoS One, 6(6), e14821 10.1371/journal.pone.0014821 21731603 PMC3120753

[acel13280-bib-0003] Coschigano, K. T. , Holland, A. N. , Riders, M. E. , List, E. O. , Flyvbjerg, A. , & Kopchick, J. J. (2003). Deletion, but not antagonism, of the mouse growth hormone receptor results in severely decreased body weights, insulin, and insulin‐like growth factor I levels and increased life span. Endocrinology, 144(9), 3799–3810. 10.1210/en.2003-0374 12933651

[acel13280-bib-0004] Couronne, R. , Probst, P. , & Boulesteix, A. L. (2018). Random forest versus logistic regression: A large‐scale benchmark experiment. BMC Bioinformatics, 19(1), 270 10.1186/s12859-018-2264-5 30016950 PMC6050737

[acel13280-bib-0005] de Magalhaes, J. P. , Curado, J. , & Church, G. M. (2009). Meta‐analysis of age‐related gene expression profiles identifies common signatures of aging. Bioinformatics, 25(7), 875–881. 10.1093/bioinformatics/btp073 19189975 PMC2732303

[acel13280-bib-0006] Deelen, J. , Evans, D. S. , Arking, D. E. , Tesi, N. , Nygaard, M. , Liu, X. , Wojczynski, M. K. , Biggs, M. L. , van der Spek, A. , Atzmon, G. , Ware, E. B. , Sarnowski, C. , Smith, A. V. , Seppälä, I. , Cordell, H. J. , Dose, J. , Amin, N. , Arnold, A. M. , Ayers, K. L. , … Murabito, J. M. (2019). A meta‐analysis of genome‐wide association studies identifies multiple longevity genes. Nature Communications, 10(1), 3669 10.1038/s41467-019-11558-2 PMC6694136 31413261

[acel13280-bib-0007] Dobin, A. , Davis, C. A. , Schlesinger, F. , Drenkow, J. , Zaleski, C. , Jha, S. , Batut, P. , Chaisson, M. , & Gingeras, T. R. (2013). STAR: Ultrafast universal RNA‐seq aligner. Bioinformatics, 29(1), 15–21. 10.1093/bioinformatics/bts635 23104886 PMC3530905

[acel13280-bib-0008] Fahy, G. M. , Brooke, R. T. , Watson, J. P. , Good, Z. , Vasanawala, S. S. , Maecker, H. , Leipold, M. D. , Lin, D. T. S. , Kobor, M. S. , & Horvath, S. (2019). Reversal of epigenetic aging and immunosenescent trends in humans. Aging Cell, 18(6), e13028 10.1111/acel.13028 31496122 PMC6826138

[acel13280-bib-0009] Fleischer, J. G. , Schulte, R. , Tsai, H. H. , Tyagi, S. , Ibarra, A. , Shokhirev, M. N. , Huang, L. , Hetzer, M. W. , & Navlakha, S. (2018). Predicting age from the transcriptome of human dermal fibroblasts. Genome Biology, 19(1), 221 10.1186/s13059-018-1599-6 30567591 PMC6300908

[acel13280-bib-0010] Furman, D. , Campisi, J. , Verdin, E. , Carrera‐Bastos, P. , Targ, S. , Franceschi, C. , Ferrucci, L. , Gilroy, D. W. , Fasano, A. , Miller, G. W. , Miller, A. H. , Mantovani, A. , Weyand, C. M. , Barzilai, N. , Goronzy, J. J. , Rando, T. A. , Effros, R. B. , Lucia, A. , Kleinstreuer, N. , & Slavich, G. M. (2019). Chronic inflammation in the etiology of disease across the life span. Nature Medicine, 25(12), 1822–1832. 10.1038/s41591-019-0675-0 PMC7147972 31806905

[acel13280-bib-0011] Galkin, F. , Mamoshina, P. , Aliper, A. , de Magalhaes, J. P. , Gladyshev, V. N. , & Zhavoronkov, A. (2020). Biohorology and biomarkers of aging: Current state‐of‐the‐art, challenges and opportunities. Ageing Research Reviews, 60, 101050 10.1016/j.arr.2020.101050 32272169

[acel13280-bib-0012] Gialluisi, A. , Di Castelnuovo, A. , Donati, M. B. , de Gaetano, G. , Iacoviello, Licia , & Moli‐sani Study Investigators . (2019). Machine learning approaches for the estimation of biological aging: The road ahead for population studies. Frontiers in Medicine, 6, 146 10.3389/fmed.2019.00146 31338367 PMC6626911

[acel13280-bib-0013] Glasson, S. S. , Askew, R. , Sheppard, B. , Carito, B. , Blanchet, T. , Ma, H.‐L. , Flannery, C. R. , Peluso, D. , Kanki, K. , Yang, Z. , Majumdar, M. K. , & Morris, E. A. (2005). Deletion of active ADAMTS5 prevents cartilage degradation in a murine model of osteoarthritis. Nature, 434(7033), 644–648. 10.1038/nature03369 15800624

[acel13280-bib-0014] Hakimi, P. , Yang, J. , Casadesus, G. , Massillon, D. , Tolentino‐Silva, F. , Nye, C. K. , Cabrera, M. E. , Hagen, D. R. , Utter, C. B. , Baghdy, Y. , Johnson, D. H. , Wilson, D. L. , Kirwan, J. P. , Kalhan, S. C. , & Hanson, R. W. (2007). Overexpression of the cytosolic form of phosphoenolpyruvate carboxykinase (GTP) in skeletal muscle repatterns energy metabolism in the mouse. Journal of Biological Chemistry, 282(45), 32844–32855. 10.1074/jbc.M706127200 PMC4484620 17716967

[acel13280-bib-0015] Hannum, G. , Guinney, J. , Zhao, L. , Zhang, L. I. , Hughes, G. , Sadda, S. V. , Klotzle, B. , Bibikova, M. , Fan, J.‐B. , Gao, Y. , Deconde, R. , Chen, M. , Rajapakse, I. , Friend, S. , Ideker, T. , & Zhang, K. (2013). Genome‐wide methylation profiles reveal quantitative views of human aging rates. Molecular Cell, 49(2), 359–367. 10.1016/j.molcel.2012.10.016 23177740 PMC3780611

[acel13280-bib-0016] Hedbacker, K. , Birsoy, K. , Wysocki, R. W. , Asilmaz, E. , Ahima, R. S. , Farooqi, I. S. , & Friedman, J. M. (2010). Antidiabetic effects of IGFBP2, a leptin‐regulated gene. Cell Metabolism, 11(1), 11–22. 10.1016/j.cmet.2009.11.007 20074524

[acel13280-bib-0017] Horvath, S. (2013). DNA methylation age of human tissues and cell types. Genome Biology, 14(10), R115 10.1186/gb-2013-14-10-r115 24138928 PMC4015143

[acel13280-bib-0018] Horvath, S. , & Raj, K. (2018). DNA methylation‐based biomarkers and the epigenetic clock theory of ageing. Nature Reviews Genetics, 19(6), 371–384. 10.1038/s41576-018-0004-3 29643443

[acel13280-bib-0019] Huan, T. , Chen, G. , Liu, C. , Bhattacharya, A. , Rong, J. , Chen, B. H. , Seshadri, S. , Tanriverdi, K. , Freedman, J. E. , Larson, M. G. , Murabito, J. M. , & Levy, D. (2018). Age‐associated microRNA expression in human peripheral blood is associated with all‐cause mortality and age‐related traits. Aging Cell, 17(1), e12687 10.1111/acel.12687 PMC5770777 29044988

[acel13280-bib-0020] Hulsen, T. , de Vlieg, J. , & Alkema, W. (2008). BioVenn ‐ a web application for the comparison and visualization of biological lists using area‐proportional Venn diagrams. BMC Genomics, 9, 488 10.1186/1471-2164-9-488 18925949 PMC2584113

[acel13280-bib-0021] Jo, T. , Nho, K. , & Saykin, A. J. (2019). Deep learning in Alzheimer's disease: Diagnostic classification and prognostic prediction using neuroimaging data. Frontiers in Aging Neuroscience, 11, 220 10.3389/fnagi.2019.00220 31481890 PMC6710444

[acel13280-bib-0022] Johnson, A. A. , Shokhirev, M. N. , & Shoshitaishvili, B. (2019). Revamping the evolutionary theories of aging. Ageing Research Reviews, 55, 100947 10.1016/j.arr.2019.100947 31449890

[acel13280-bib-0023] Johnson, A. A. , Shokhirev, M. N. , Wyss‐Coray, T. , & Lehallier, B. (2020). Systematic review and analysis of human proteomics aging studies unveils a novel proteomic aging clock and identifies key processes that change with age. Ageing Research Reviews, 60, 101070 10.1016/j.arr.2020.101070 32311500

[acel13280-bib-0024] Kuhn, M. (2008). Building predictive models in r using the caret package. Journal of Statistical Software, 28(5), 1–26.27774042

[acel13280-bib-0025] Kunkle, B. W. , Grenier‐Boley, B. , Sims, R. , Bis, J. C. , Damotte, V. , Naj, A. C. , Boland, A. , Vronskaya, M. , van der Lee, S. J. , Amlie‐Wolf, A. , Bellenguez, C. , Frizatti, A. , Chouraki, V. , Martin, E. R. , Sleegers, K. , Badarinarayan, N. , Jakobsdottir, J. , Hamilton‐Nelson, K. L. , Moreno‐Grau, S. , …Environmental Risk for Alzheimer’s Disease Consortium . (2019). Genetic meta‐analysis of diagnosed Alzheimer's disease identifies new risk loci and implicates Abeta, tau, immunity and lipid processing. Nature Genetics, 51(3), 414–430. 10.1038/s41588-019-0358-2 30820047 PMC6463297

[acel13280-bib-0026] LaRocca, T. J. , Cavalier, A. N. , & Wahl, D. (2020). Repetitive elements as a transcriptomic marker of aging: Evidence in multiple datasets and models. Aging Cell, 19(7), 1–6. 10.1111/acel.13167 PMC7412685 32500641

[acel13280-bib-0027] Lee, J. K. H. , Lam, G. K. L. , Shin, T. , Kim, J. , Krishnan, A. , Greenberg, D. P. , & Chit, A. (2018). Efficacy and effectiveness of high‐dose versus standard‐dose influenza vaccination for older adults: a systematic review and meta‐analysis. Expert Rev Vaccines, 17(5), 435–443. 10.1080/14760584.2018.1471989 29715054

[acel13280-bib-0028] Leek, J. T. , Johnson, W. E. , Parker, H. S. , Jaffe, A. E. , & Storey, J. D. (2012). The sva package for removing batch effects and other unwanted variation in high‐throughput experiments. Bioinformatics, 28(6), 882–883. 10.1093/bioinformatics/bts034 22257669 PMC3307112

[acel13280-bib-0029] Lehallier, B. , Shokhirev, M. N. , Wyss‐Coray, T. , & Johnson, A. A. (2020). Data mining of human plasma proteins generates a multitude of highly predictive aging clocks that reflect different aspects of aging. Aging Cell, e13256, 19(11), 1–19. 10.1111/acel.13256 33031577 PMC7681068

[acel13280-bib-0030] Leinonen, R. , Sugawara, H. , Shumway, M. , & International Nucleotide Sequence Database Collaboration . (2011). The sequence read archive. Nucleic Acids Research, 39(Database issue), D19–D21. 10.1093/nar/gkq1019 21062823 PMC3013647

[acel13280-bib-0031] Levine, M. E. , Lu, A. T. , Quach, A. , Chen, B. H. , Assimes, T. L. , Bandinelli, S. , Hou, L. , Baccarelli, A. A. , Stewart, J. D. , Li, Y. , Whitsel, E. A. , Wilson, J. G. , Reiner, A. P. , Aviv, A. , Lohman, K. , Liu, Y. , Ferrucci, L. , & Horvath, S. (2018). An epigenetic biomarker of aging for lifespan and healthspan. Aging (Albany NY), 10(4), 573–591. 10.18632/aging.101414 29676998 PMC5940111

[acel13280-bib-0032] Liao, Y. , Wang, J. , Jaehnig, E. J. , Shi, Z. , & Zhang, B. (2019). WebGestalt 2019: gene set analysis toolkit with revamped UIs and APIs. Nucleic Acids Research, 47(W1), W199–W205. 10.1093/nar/gkz401 31114916 PMC6602449

[acel13280-bib-0033] Lu, A. T. , Quach, A. , Wilson, J. G. , Reiner, A. P. , Aviv, A. , Raj, K. , Hou, L. , Baccarelli, A. A. , Li, Y. , Stewart, J. D. , Whitsel, E. A. , Assimes, T. L. , Ferrucci, L. , & Horvath, S. (2019). DNA methylation GrimAge strongly predicts lifespan and healthspan. Aging (Albany NY), 11(2), 303–327. 10.18632/aging.101684 30669119 PMC6366976

[acel13280-bib-0034] Mamoshina, P. , Volosnikova, M. , Ozerov, I. V. , Putin, E. , Skibina, E. , Cortese, F. , & Zhavoronkov, A. (2018). Machine learning on human muscle transcriptomic data for biomarker discovery and tissue‐specific drug target identification. Front Genet, 9, 242 10.3389/fgene.2018.00242 30050560 PMC6052089

[acel13280-bib-0035] Marschallinger, J. , Iram, T. , Zardeneta, M. , Lee, S. E. , Lehallier, B. , Haney, M. S. , Pluvinage, J. V. , Mathur, V. , Hahn, O. , Morgens, D. W. , Kim, J. , Tevini, J. , Felder, T. K. , Wolinski, H. , Bertozzi, C. R. , Bassik, M. C. , Aigner, L. , & Wyss‐Coray, T. (2020). Lipid‐droplet‐accumulating microglia represent a dysfunctional and proinflammatory state in the aging brain. Nature Neuroscience, 23(2), 194–208. 10.1038/s41593-019-0566-1 31959936 PMC7595134

[acel13280-bib-0036] Muro, A. F. , Chauhan, A. K. , Gajovic, S. , Iaconcig, A. , Porro, F. , Stanta, G. , & Baralle, F. E. (2003). Regulated splicing of the fibronectin EDA exon is essential for proper skin wound healing and normal lifespan. Journal of Cell Biology, 162(1), 149–160. 10.1083/jcb.200212079 PMC2172721 12847088

[acel13280-bib-0037] Peters, M. J. , Joehanes, R. , Pilling, L. C. , Schurmann, C. , Conneely, K. N. , Powell, J. , Reinmaa, E. , Sutphin, G. L. , Zhernakova, A. , Schramm, K. , Wilson, Y. A. , Kobes, S. , Tukiainen, T. , Ramos, Y. F. , Göring, H. H. H. , Fornage, M. , Liu, Y. , Gharib, S. A. , Stranger, B. E. , … Johnson, A. D. (2015). The transcriptional landscape of age in human peripheral blood. Nature Communications, 6, 8570 10.1038/ncomms9570 PMC4639797 26490707

[acel13280-bib-0038] Robinson, M. D. , McCarthy, D. J. , & Smyth, G. K. (2010). edgeR: A Bioconductor package for differential expression analysis of digital gene expression data. Bioinformatics, 26(1), 139–140. 10.1093/bioinformatics/btp616 19910308 PMC2796818

[acel13280-bib-0039] Schaum, N. , Lehallier, B. , Hahn, O. , Pálovics, R. , Hosseinzadeh, S. , Lee, S. E. , Sit, R. , Lee, D. P. , Losada, P. M. , Zardeneta, M. E. , Fehlmann, T. , Webber, J. T. , McGeever, A. , Calcuttawala, K. , Zhang, H. , Berdnik, D. , Mathur, V. , Tan, W. , Zee, A. , … Wyss‐Coray, T. (2020). Ageing hallmarks exhibit organ‐specific temporal signatures. Nature, 583(7817), 596–602. 10.1038/s41586-020-2499-y 32669715 PMC7757734

[acel13280-bib-0040] Schultz, M. B. , Kane, A. E. , Mitchell, S. J. , MacArthur, M. R. , Warner, E. , Vogel, D. S. , Mitchell, J. R. , Howlett, S. E. , Bonkowski, M. S. , & Sinclair, D. A. (2020). Age and life expectancy clocks based on machine learning analysis of mouse frailty. Nature Communications, 11(1), 4618 10.1038/s41467-020-18446-0 PMC7492249 32934233

[acel13280-bib-0041] Shavlakadze, T. , Morris, M. , Fang, J. , Wang, S. X. , Zhu, J. , Zhou, W. , Tse, H. W. , Mondragon‐Gonzalez, R. , Roma, G. , & Glass, D. J. (2019). Age‐related gene expression signature in rats demonstrate early, late, and linear transcriptional changes from multiple tissues. Cell Reports, 28(12), 3263–3273 e3263. 10.1016/j.celrep.2019.08.043 31533046

[acel13280-bib-0042] Smith, H. J. , Sharma, A. , & Mair, W. B. (2020). Metabolic communication and healthy aging: Where should we focus our energy? Developmental Cell, 54(2), 196–211. 10.1016/j.devcel.2020.06.011 32619405 PMC8168458

[acel13280-bib-0043] Smith, L. K. , He, Y. , Park, J. S. , Bieri, G. , Snethlage, C. E. , Lin, K. , Gontier, G. , Wabl, R. , Plambeck, K. E. , Udeochu, J. , Wheatley, E. G. , Bouchard, J. , Eggel, A. , Narasimha, R. , Grant, J. L. , Luo, J. , Wyss‐Coray, T. , & Villeda, S. A. (2015). beta2‐microglobulin is a systemic pro‐aging factor that impairs cognitive function and neurogenesis. Nature Medicine, 21(8), 932–937. 10.1038/nm.3898 PMC4529371 26147761

[acel13280-bib-0044] The Gene Ontology Consortium . (2019). The Gene Ontology Resource: 20 years and still GOing strong. Nucleic Acids Research, 47(D1), D330–D338. 10.1093/nar/gky1055 30395331 PMC6323945

[acel13280-bib-0045] Touw, W. G. , Bayjanov, J. R. , Overmars, L. , Backus, L. , Boekhorst, J. , Wels, M. , & van Hijum, S. A. (2013). Data mining in the Life Sciences with Random Forest: a walk in the park or lost in the jungle? Briefings in Bioinformatics, 14(3), 315–326. 10.1093/bib/bbs034 22786785 PMC3659301

[acel13280-bib-0046] Vafaie, F. , Yin, H. , O'Neil, C. , Nong, Z. , Watson, A. , Arpino, J.‐M. , Chu, M. W. A. , Wayne Holdsworth, D. , Gros, R. , & Pickering, J. G. (2014). Collagenase‐resistant collagen promotes mouse aging and vascular cell senescence. Aging Cell, 13(1), 121–130. 10.1111/acel.12155 23957394 PMC4326859

[acel13280-bib-0047] Valdes, A. M. , Glass, D. , & Spector, T. D. (2013). Omics technologies and the study of human ageing. Nature Reviews Genetics, 14(9), 601–607. 10.1038/nrg3553 23938363

[acel13280-bib-0048] Voisin, S. , Harvey, N. R. , Haupt, L. M. , Griffiths, L. R. , Ashton, K. J. , Coffey, V. G. , Doering, T. M. , Thompson, J.‐L. , Benedict, C. , Cedernaes, J. , Lindholm, M. E. , Craig, J. M. , Rowlands, D. S. , Sharples, A. P. , Horvath, S. , & Eynon, N. (2020). An epigenetic clock for human skeletal muscle. Journal of Cachexia, Sarcopenia and Muscle, 11(4), 887–898. 10.1002/jcsm.12556 PMC7432573 32067420

[acel13280-bib-0049] Wang, X. , Chrysovergis, K. , Kosak, J. , Kissling, G. , Streicker, M. , Moser, G. , Li, R. , & Eling, T. E. (2014). hNAG‐1 increases lifespan by regulating energy metabolism and insulin/IGF‐1/mTOR signaling. Aging (Albany NY), 6(8), 690–704. 10.18632/aging.100687 25239873 PMC4169862

[acel13280-bib-0050] Zeng, L. , Yang, J. , Peng, S. , Zhu, J. , Zhang, B. , Suh, Y. , & Tu, Z. (2020). Transcriptome analysis reveals the difference between "healthy" and "common" aging and their connection with age‐related diseases. Aging Cell, 19(3), e13121 10.1111/acel.13121 32077223 PMC7059150

[acel13280-bib-0051] Zhang, S. H. , Reddick, R. L. , Piedrahita, J. A. , & Maeda, N. (1992). Spontaneous hypercholesterolemia and arterial lesions in mice lacking apolipoprotein E. Science, 258(5081), 468–471. 10.1126/science.1411543 1411543

[acel13280-bib-0052] Zhang, Y. , Proenca, R. , Maffei, M. , Barone, M. , Leopold, L. , & Friedman, J. M. (1994). Positional cloning of the mouse obese gene and its human homologue. Nature, 372(6505), 425–432. 10.1038/372425a0 7984236

[acel13280-bib-0053] Zhavoronkov, A. , Li, R. , Ma, C. , & Mamoshina, P. (2019). Deep biomarkers of aging and longevity: from research to applications. Aging (Albany NY), 11(22), 10771–10780. 10.18632/aging.102475 31767810 PMC6914424

