# Supplementary figures and images for: Modeling the human aging transcriptome across tissues, health status, and sex
Source: Aging Cell. 2020 Dec 18;20(1):e13280. doi: 10.1111/acel.13280 (PMC7811842; doi:10.1111/acel.13280)

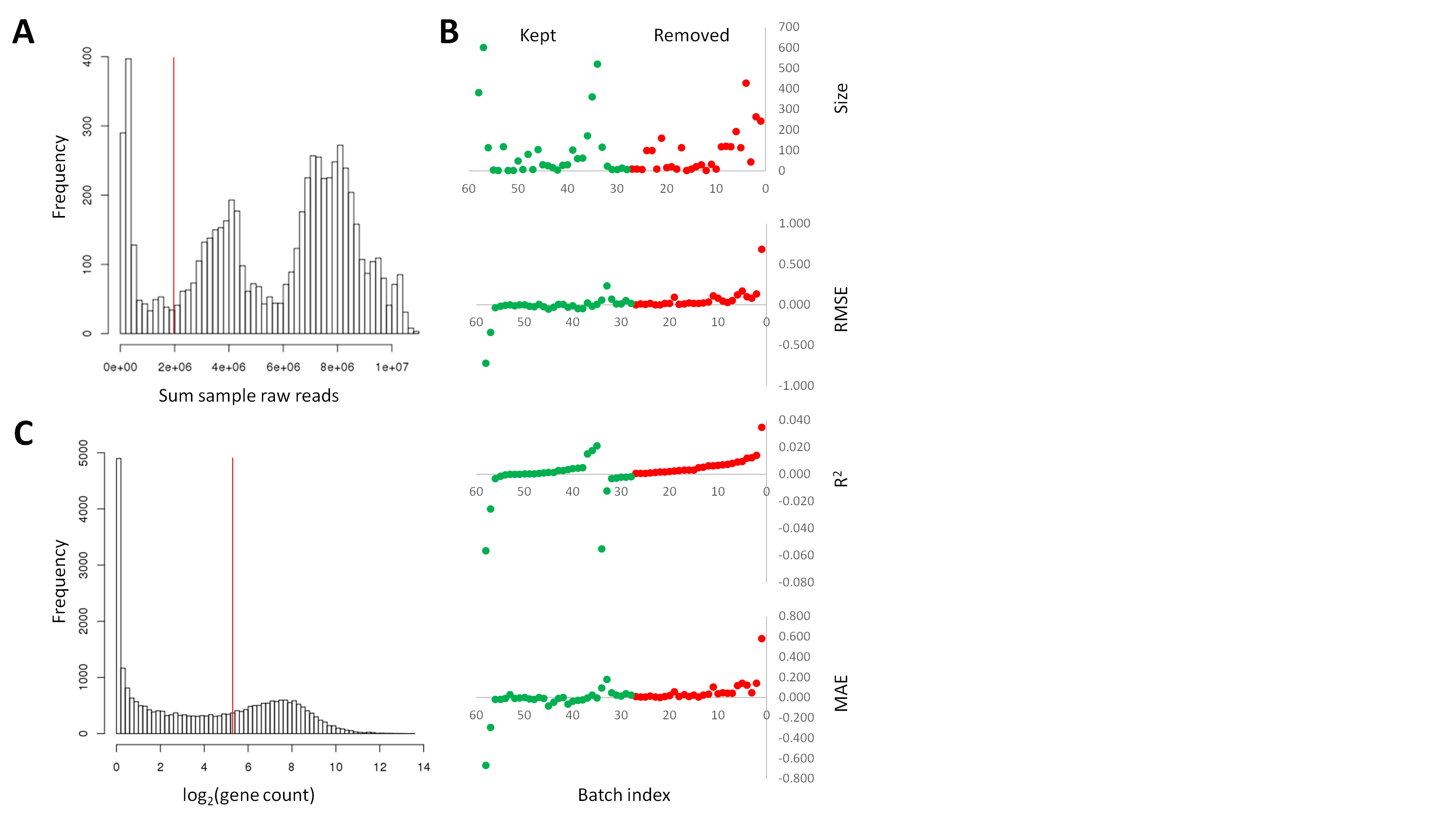

Supplement: Supplementary file 1 — Figure S1 [file ACEL-20-e13280-s001.TIF]

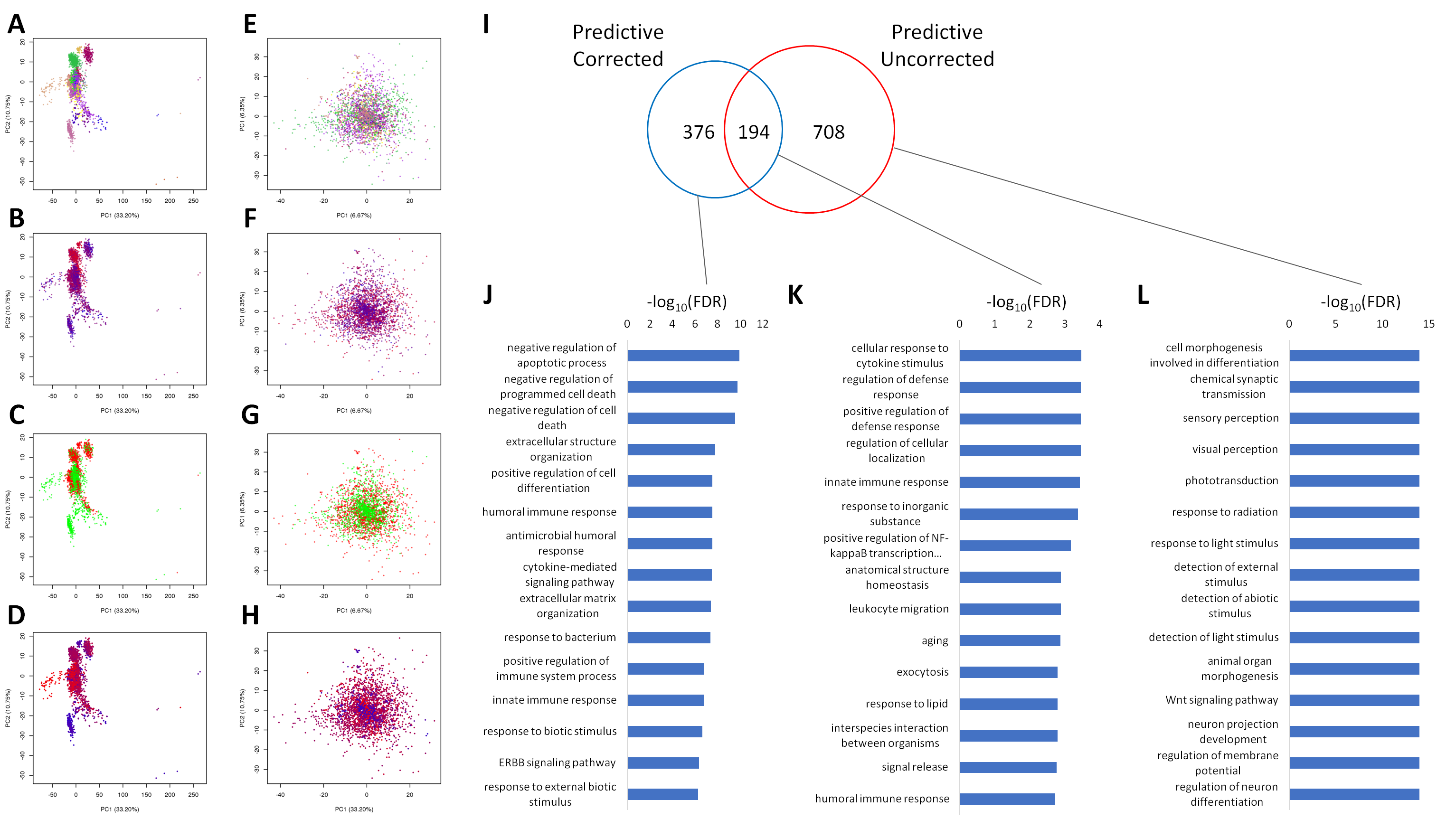

Supplement: Supplementary file 2 — Figure S2 [file ACEL-20-e13280-s002.TIF]

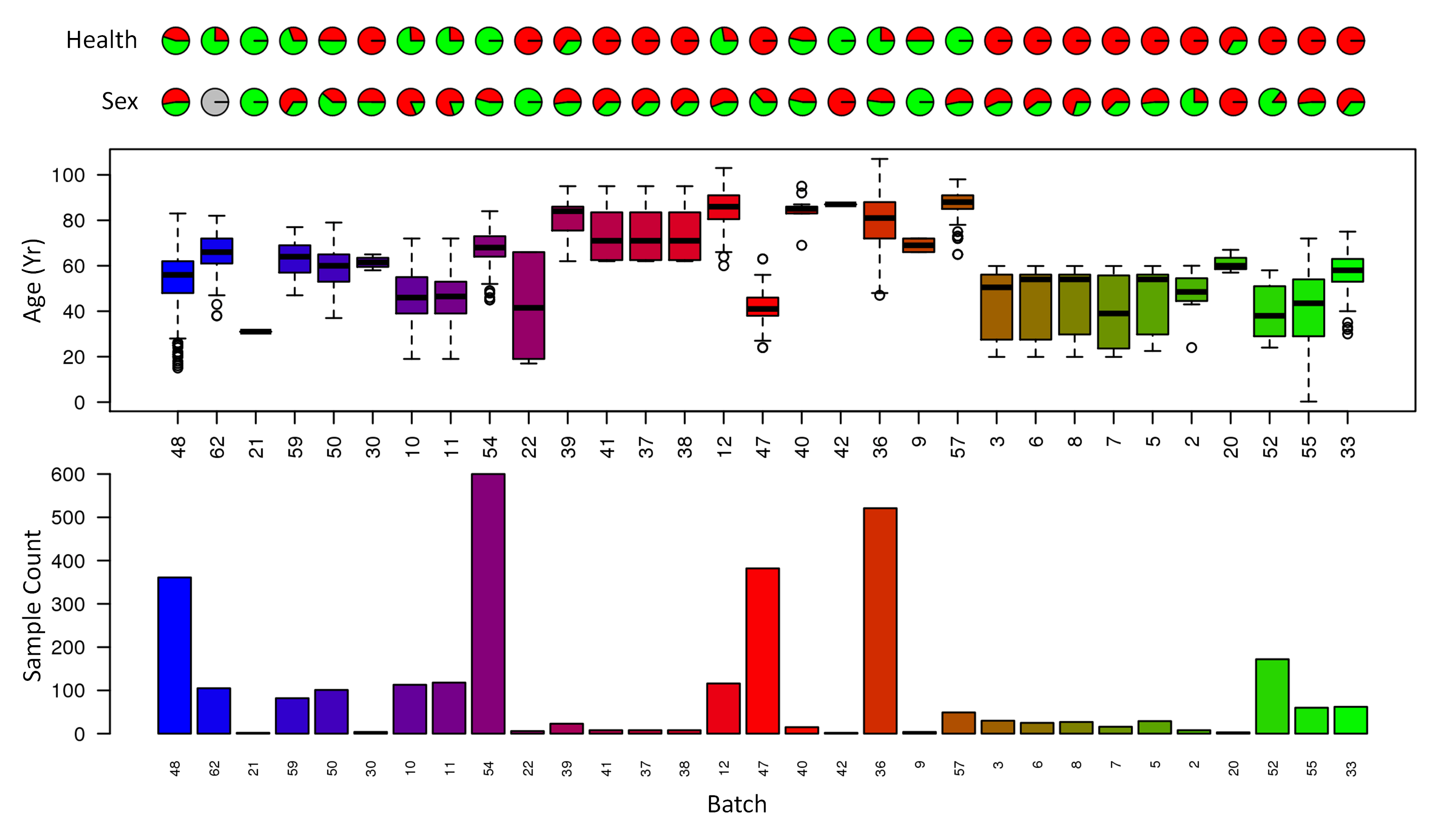

Supplement: Supplementary file 3 — Figure S3 [file ACEL-20-e13280-s003.TIF]

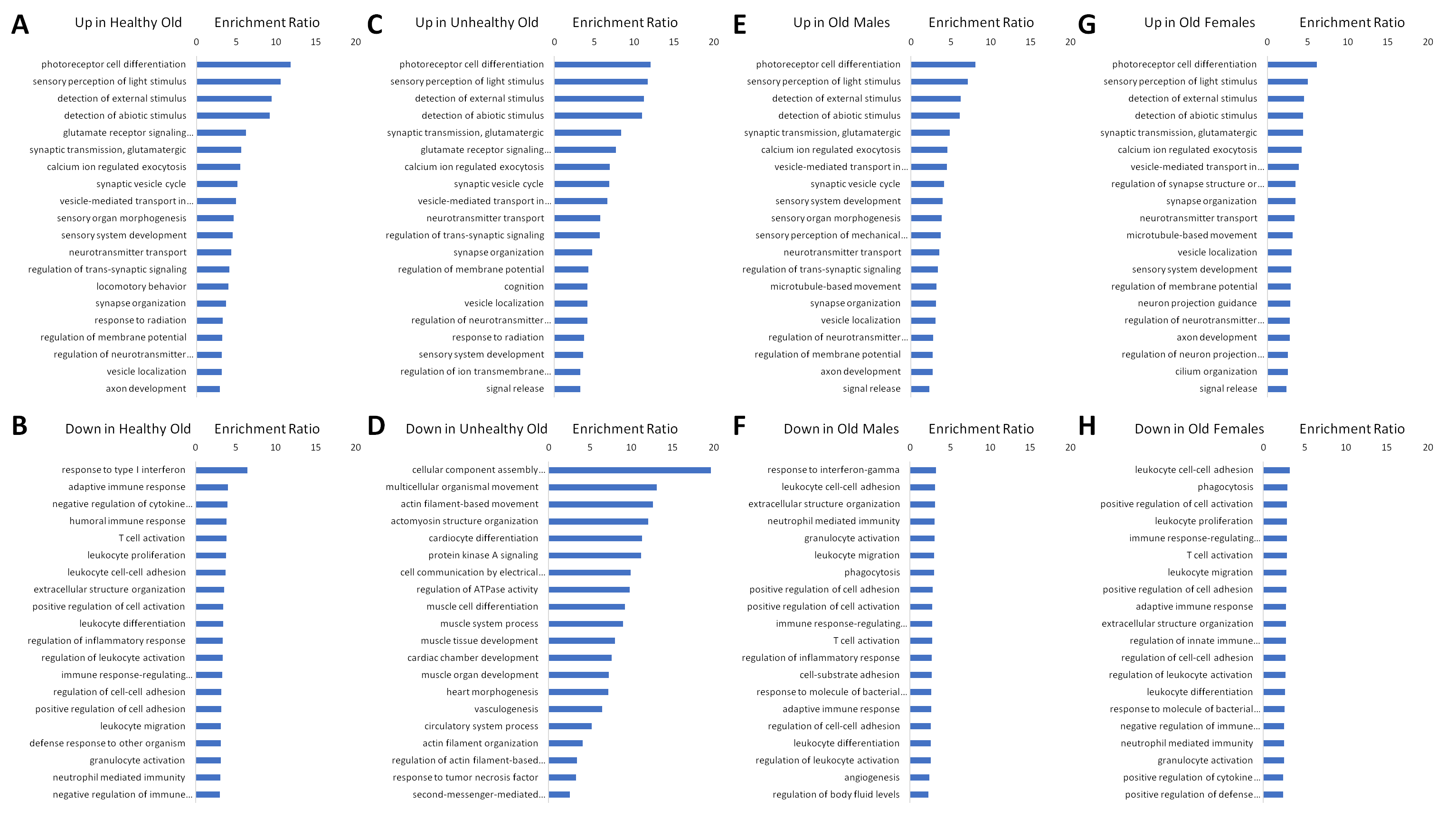

Supplement: Supplementary file 4 — Figure S4 [file ACEL-20-e13280-s004.TIF]

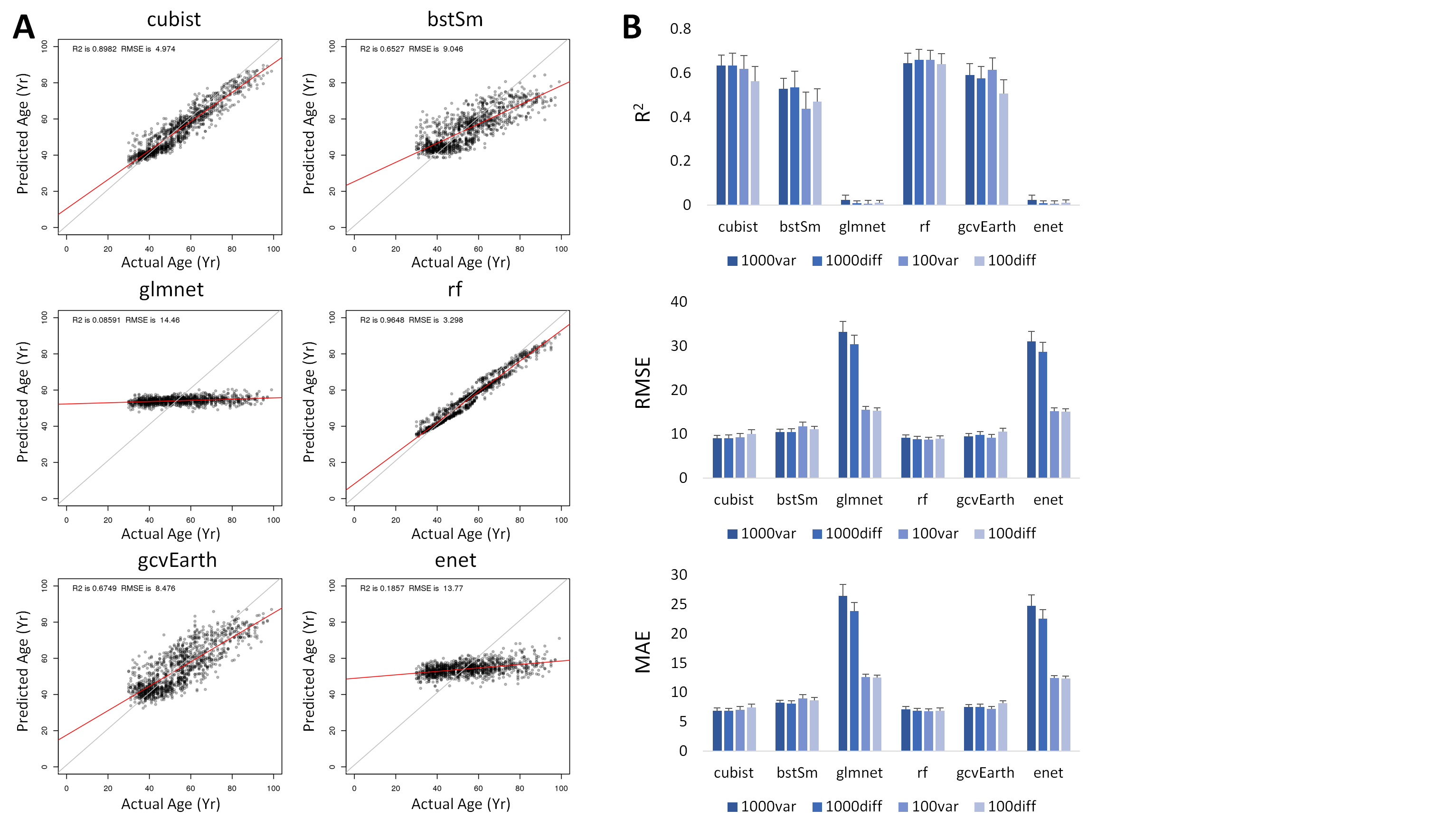

Supplement: Supplementary file 5 — Figure S5 [file ACEL-20-e13280-s005.TIF]

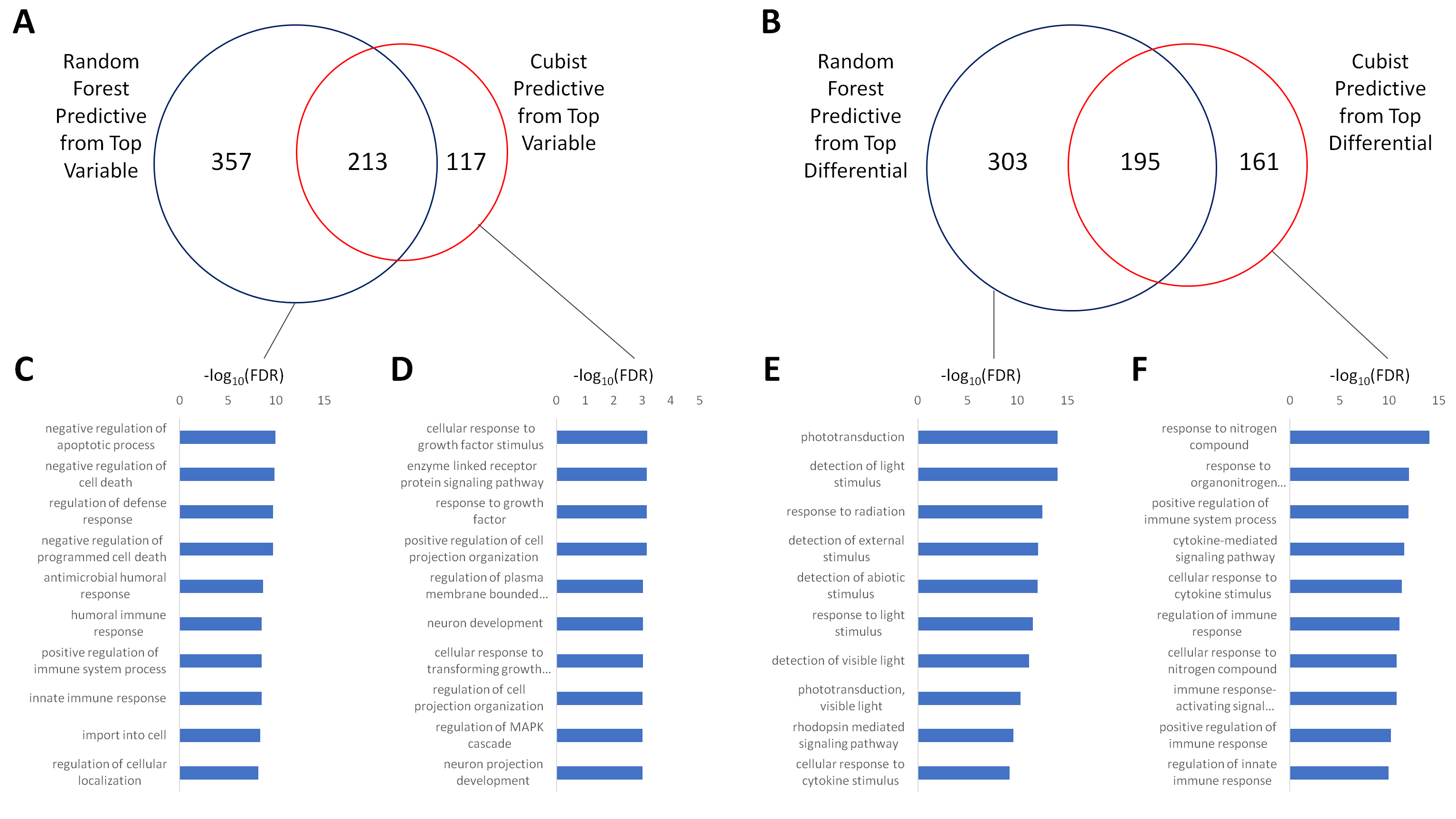

Supplement: Supplementary file 6 — Figure S6 [file ACEL-20-e13280-s006.TIF]

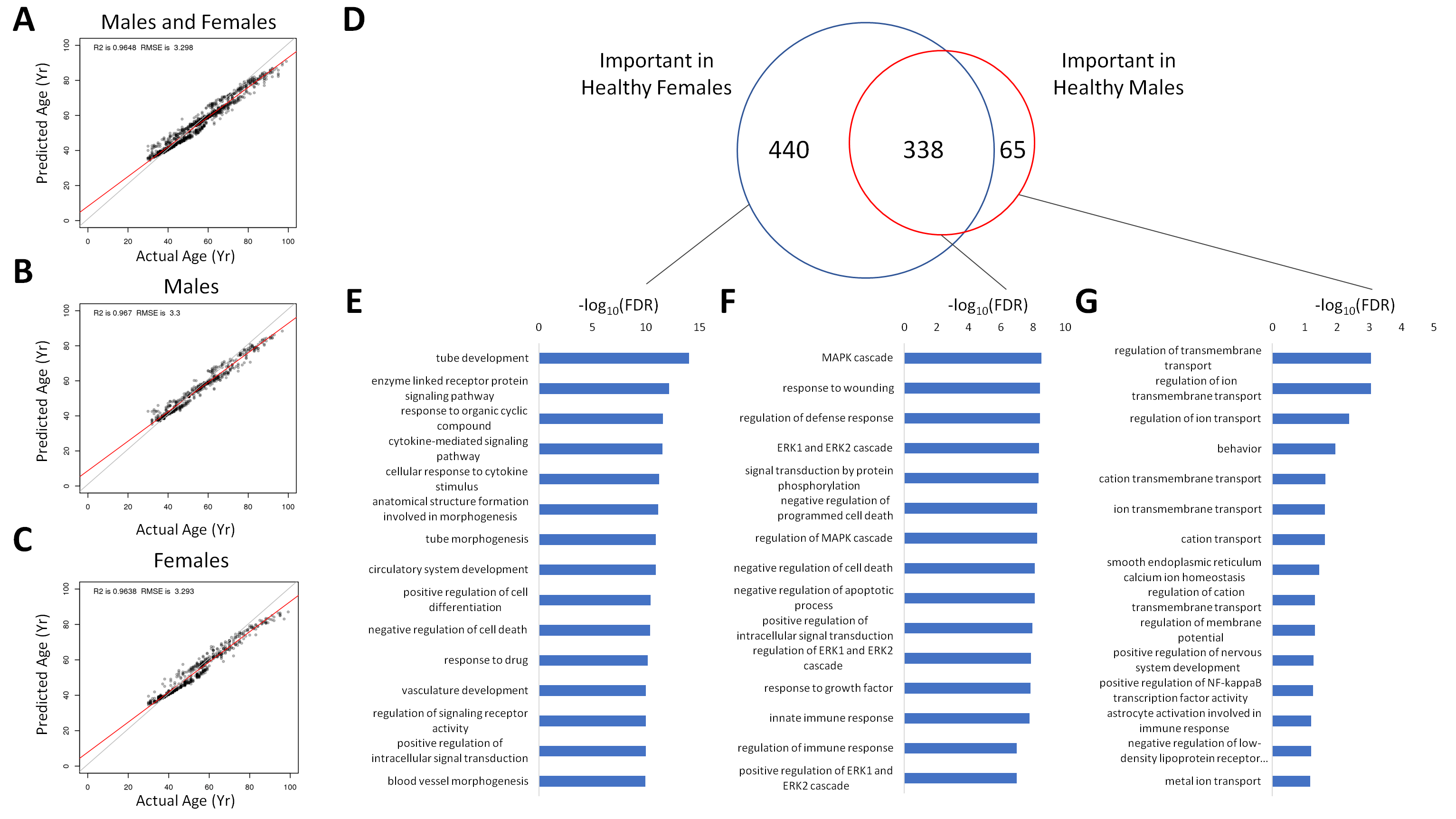

Supplement: Supplementary file 7 — Figure S7 [file ACEL-20-e13280-s007.TIF]
