# Supplementary material for: Modeling the human aging transcriptome across tissues, health status, and sex
Source: Aging Cell. 2020 Dec 18;20(1):e13280. doi: 10.1111/acel.13280 (PMC7811842; doi:10.1111/acel.13280)
Supplement: Supplementary file 9 — Supplementary Material [file ACEL-20-e13280-s009.docx]

**Supplementary Figure Legends**

Figure S1. Filtering samples, batches, and genes. A) Distribution of sample total counts with a cutoff of 2 million. B) Table describing relative change in fit when each batch is removed. Green-highlighted batches were used in downstream analyses while those in red were removed. C) Distribution of gene counts and threshold used to remove low-expressing genes.

Figure S2. Effect of batch correction on model accuracy. A-H) PCA showing clustering of batches prior to (A-D) and after (E-H) batch correction. A, E) Coloring by batch. B, F) Coloring by age (red = old). C, G) Coloring by health status (green = healthy). D, H) Coloring by total raw count (red = high). I) Venn diagram showing overlap in predictive genes for the model trained on batch-corrected and uncorrected data. J-L) Top 15 Gene Ontology categories identified using network topology based enrichment analysis on subsets of important genes predictive in corrected-only data (J), both corrected and uncorrected data (K), or uncorrected-only data (L).

Figure S3. Batch statistics for health status, sex, age, and sample count. For health status, green = healthy and red = unhealthy. For sex status, red = males, green = females, and gray = not reported.

Figure S4. Health and sex status impact which biological processes are altered with age. Differential expression analyses were performed to identify which genes change with age for different human cohorts. For each set of genes identified, an over-representation analysis was performed and the top 20 Gene Ontological Biological Process enrichment terms are shown. Results for genes that either increase or decrease with age are shown for healthy adults (A, B), unhealthy adults (C, D), healthy males (E, F), and healthy females (G, H).

Figure S5. Testing different machine learning models for the prediction of human age. We compared cubist, glmnet, gcvEarth, bstSm, rf, and enet models implemented in the caret package. A) Predictions of healthy adults for these model types using the top 1,000 variable genes for training. B) Summary of R^2^ values, root mean squared errors (RMSEs), and mean absolute errors (MAEs) for each model trained using different sets of variable or differential genes.

Figure S6. Comparison of predictive genes between random forest and cubist regression models. A, B) Venn diagrams showing overlap of important predictive genes (score > 1) for random forest and cubist models trained on the top 1,000 variable (A) and top 1,000 differential (B) genes from healthy samples. C-F) Network topology based enrichment analyses of predictive genes trained on top 1,000 variable genes (C, D) or top 1,000 differential genes (E, F). Genes predictive only for the random forest model (C, E) or the cubist model (D, F) were used as input for enrichment and the top 10 significant terms were plotted as bar graphs.

Figure S7. Sex affects aging clocks. A) Sex-independent model fit on healthy adults, including both males and females. B) Model fit on only male healthy adults. C) Model fit on only female healthy adults. D) Overlap of predictive genes (score > 1) from the healthy female and healthy male models. E) Network topology-based analysis of predictive genes unique to the healthy female model. F) Network topology-based analysis of predictive genes common to healthy female and male models. G) Network topology-based analysis of predictive genes unique to the healthy male model. For each analysis (E-G), the top 15 significant terms are shown.
